# Endothelial Cells Use a Formin-Dependent Phagocytosis-Like Process to Internalize the Bacterium *Listeria monocytogenes*

**DOI:** 10.1371/journal.ppat.1005603

**Published:** 2016-05-06

**Authors:** Michelle Rengarajan, Arnold Hayer, Julie A. Theriot

**Affiliations:** 1 Department of Biochemistry, Stanford University School of Medicine, Stanford, California, United States of America; 2 Department of Chemical and Systems Biology, Stanford University School of Medicine, Stanford, California, United States of America; 3 Department of Microbiology and Immunology, Stanford University School of Medicine, Stanford, California, United States of America; 4 Howard Hughes Medical Institute, Stanford University School of Medicine, Stanford, California, United States of America; University of Michigan Medical School, UNITED STATES

## Abstract

Vascular endothelial cells act as gatekeepers that protect underlying tissue from blood-borne toxins and pathogens. Nevertheless, endothelial cells are able to internalize large fibrin clots and apoptotic debris from the bloodstream, although the precise mechanism of such phagocytosis-like uptake is unknown. We show that cultured primary human endothelial cells (HUVEC) internalize both pathogenic and non-pathogenic *Listeria* bacteria comparably, in a phagocytosis-like process. In contrast with previously studied host cell types, including intestinal epithelial cells and hepatocytes, we find that endothelial internalization of *Listeria* is independent of all known pathogenic bacterial surface proteins. Consequently, we exploited the internalization and intracellular replication of *L*. *monocytogenes* to identify distinct host cell factors that regulate phagocytosis-like uptake in HUVEC. Using siRNA screening and subsequent genetic and pharmacologic perturbations, we determined that endothelial infectivity was modulated by cytoskeletal proteins that normally modulate global architectural changes, including phosphoinositide-3-kinase, focal adhesions, and the small GTPase Rho. We found that Rho kinase (ROCK) is acutely necessary for adhesion of *Listeria* to endothelial cells, whereas the actin-nucleating formins FHOD1 and FMNL3 specifically regulate internalization of bacteria as well as inert beads, demonstrating that formins regulate endothelial phagocytosis-like uptake independent of the specific cargo. Finally, we found that neither ROCK nor formins were required for macrophage phagocytosis of *L*. *monocytogenes*, suggesting that endothelial cells have distinct requirements for bacterial internalization from those of classical professional phagocytes. Our results identify a novel pathway for *L*. *monocytogenes* uptake by human host cells, indicating that this wily pathogen can invade a variety of tissues by using a surprisingly diverse suite of distinct uptake mechanisms that operate differentially in different host cell types.

## Author Summary

Vascular endothelial cells, which line the lumen of blood vessels, are conventionally viewed as a restrictive barrier that protects underlying tissue from blood-borne toxins and pathogens. Nonetheless, even highly restrictive endothelial cells can internalize micron-sized objects, such as blood clots, raising the question of how such phagocytosis-like uptake occurs, and whether it is mechanistically distinct from classical phagocytic pathways. We found that the pathogenic bacterium *Listeria monocytogenes*, which must overcome the endothelial barrier to access underlying tissue, can be taken up by primary endothelial cells (HUVEC) in culture. We exploited this ability to identify molecular regulators of such phagocytosis-like uptake. We found that the formin family of actin nucleators drives such uptake, whereas these proteins did not have a significant role in phagocytosis of *L*. *monocytogenes* by macrophages. Thus, our data suggest that endothelial cells and macrophages use distinct phagocytosis-like pathways to internalize *L*. *monocytogenes*. Perturbations of the regulatory proteins that we have identified here should allow for dissection of the normal physiological functions of endothelial phagocytosis-like uptake, as well as its therapeutic potential in diverse roles such as clot resolution and drug delivery.

## Introduction

Vascular endothelial cells prevent free movement of material from the bloodstream into underlying tissues by tight regulation of cellular internalization pathways and robust cell-cell junctions. Nonetheless, in a process termed “angiophagy”, endothelial cells lining small-diameter capillaries in the brain, heart, lung, and kidney have been shown to internalize large fibrin or cholesterol clots that are subsequently released into the underlying parenchyma[1,2]. Furthermore, light and electron microscopy studies have established that liver endothelial cells can internalize apoptotic particles and latex beads *in situ*[3,4]. While this phenomenon is well documented, its molecular regulators have not been elucidated, making it difficult to establish a *bona fide* role for angiophagy *in vivo*. Additionally, it is unclear whether an endothelial phagocytosis-like process could be exploited by pathogens to access underlying tissue.

The food-borne bacterium *Listeria monocytogenes* can disseminate from the initial site of infection at the intestinal epithelium to cause meningitis, encephalitis, sepsis, and spontaneous abortion by crossing different types of vascular endothelia[5]. In fact, *L*. *monocytogenes* infects human endothelial cells themselves *in vivo*[6], but the mechanism of such infection is unknown.

*L*. *monocytogenes* can directly invade intestinal epithelial cells and hepatocytes, using the bacterial surface proteins internalin[7,8] (InlA) and InlB [9,10], respectively, which interact with host cell proteins. Once internalized into a membrane-bound compartment, *L*. *monocytogenes* expresses the pore-forming toxin listeriolysin O (LLO), which promotes release of the bacterium into the cytosol, where it replicates[11,12]. Previous studies have conflictingly suggested that invasion of endothelial cells in culture requires InlA[13], InlB[14,15] or neither[16,17]. We therefore sought to clarify whether *L*. *monocytogenes* uses internalins to invade endothelial cells or, alternatively, might use a distinct pathway, perhaps an angiophagy- or phagocytosis-like process, i.e. a process by which the bacterium does not trigger its own uptake through specific molecular recognition between its own surface proteins and those of the host cell. If *L*. *monocytogenes* exploits phagocytosis-like uptake in endothelial cells, then identifying regulators of *L*. *monocytogenes* entry may not only elucidate the myriad strategies of this model bacterial pathogen but may also provide mechanistic insight into how other large objects, such as stroke-causing clots in small-diameter blood vessels, are internalized by endothelial cells.

We examined *L*. *monocytogenes* infection in human umbilical vein endothelial cells (HUVEC), as these are human primary cells that are amenable to physical and genetic perturbation. We found that invasion was independent of pathogenic bacterial factors, suggesting that *L*. *monocytogenes* does indeed exploit a phagocytosis-like process for entry. We perturbed host cell signaling to identify specific regulators of such entry and determined that adhesion of *L*. *monocytogenes* to HUVEC requires the activity of the Rho GTPase effector kinase ROCK, and that efficiency of subsequent internalization was modulated by signaling from cell-substrate adhesions and by the formin family of actin nucleators. Furthermore, we found that these same regulators modulate phagocytosis-like uptake of non-pathogenic bacteria by HUVEC, but do not dramatically affect macrophage phagocytosis of *L*. *monocytogenes*. Our results demonstrate that endothelial cells internalize *L*. *monocytogenes* using a mechanism that is distinct from that employed by epithelial cells, hepatocytes, or professional phagocytes. Furthermore, endothelial phagocytosis-like uptake may be a previously unappreciated mechanism for systemic spread of pathogenic bacteria and viruses and for modulation of traffic from the bloodstream to the underlying parenchyma.

## Results

### Uptake of *L*. *monocytogenes* by HUVEC does not require specific bacterial pathogenic factors

A number of cell types, including PtK2[18], MDCK[19,20], mouse embryonic fibroblasts[21] and L2 cells[22], can tolerate exposure to high titers of *L*. *monocytogenes* (>100 bacteria per host cell) in culture; however, we found that exposing HUVEC to *L*. *monocytogenes* under such conditions resulted in dramatic and highly variable HUVEC death (S1A–S1D Fig). Neither the closely related bacterium *Listeria innocua*, which lacks the pathogenic apparatus of *L*. *monocytogenes*[23], nor an *L*. *monocytogenes* strain lacking the pore-forming toxin LLO (*hly* mutant, JAT314) caused HUVEC death (S1D Fig). Indeed, purified 6-His-LLO[24,25] induced early HUVEC death at low concentrations (S1F Fig). Notably, monocyte-like U937 cells did not display increased death in response to either *L*. *monocytogenes* or to purified 6-His-LLO (S1E and S1G Fig). These data collectively suggest that HUVEC are particularly sensitive to LLO and that extracellular LLO causes HUVEC death during initial exposure to high bacterial titers in culture.

**Fig 1:**
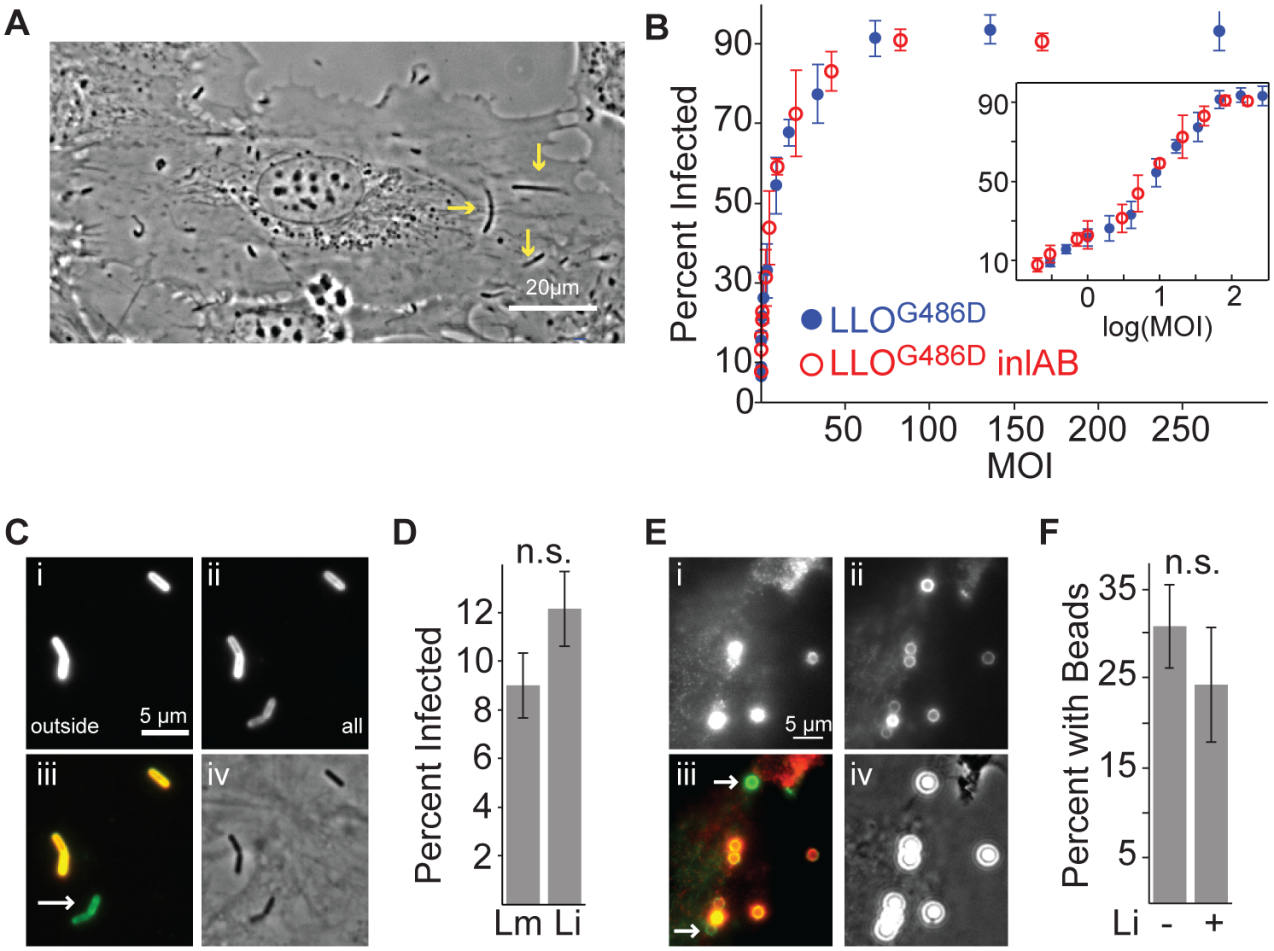
Uptake of *L*. *monocytogenes* by HUVEC is independent of bacterial factors. (**A**) Still from S1 Movie, 5 hours after infection. *L*. *monocytogenes* expressing LLO^G486D^ has invaded HUVEC, escaped the vacuole, and replicated in the cytoplasm. Bacteria are moving within the cytoplasm. Yellow arrows indicate F-actin tails associated with moving bacteria. Scale bar: 20μm. (**B**) Frequency of HUVEC with internalized bacteria as a function of the multiplicity of infection (MOI) (mean +/- standard deviation (SD), n = 4 biological replicates). HUVEC were infected with the indicated strains (JAT983 and JAT1119) and the frequency of infected HUVEC was determined by microscopy 8 hours after infection. Inset shows the same data with MOI on a log scale. (**C**, **D**) Efficient uptake of *L*. *innocua* by HUVEC. HUVEC were infected with comparable loads of *L*. *innocua* (JAT638, MOI: 11) or *L*. *monocytogenes* LLO^G486D^ (JAT745, MOI: 8). Inside/outside staining was used to determine whether bacteria were internalized. (**C**) (i) Extracellular bacteria (labeled before permeabilization of HUVEC) (ii) Extracellular and intracellular bacteria (labeled after permabilization of HUVEC) (iii) In overlay, extracellular bacteria are yellow and intracellular bacteria are green. Arrow indicates intracellular bacterium. (iv) Phase-contrast image of the same region. Scale bars: 5μm. (**D**) Frequency of HUVEC with internalized bacteria (mean +/- SD, n = 3 biological replicates). Lm = *L*. *monocytogenes*, Li = *L*. *innocua*. P-value (unpaired two-sided t-test) = 0.1577. (**E, F**) Efficient uptake of polystyrene beads by HUVEC. HUVEC were exposed to 2μm polystyrene beads (MOI: 10). Inside/outside staining was used to determine whether beads were internalized. (**E**) (i) Extracellular beads (ii) Extracellular and intracellular beads (iii) Overlay, in which extracellular beads are yellow and intracellular beads are green. Arrows indicate intracellular beads. (iv) Phase contrast image of the same region. Scale bars: 5μm. (**F**) Frequency of HUVEC with internalized beads in the absence (-) or presence (+) of *L*. *innocua* (mean +/- SD, n = 6 biological replicates). P-value (unpaired two-sided t-test) = 0.175. Parts C-F show representative data from 1 of 2 independent experiments.

An LLO point mutant, LLO^G486D^ (JAT745) has previously been reported to exhibit decreased hemolysis relative to the wild-type protein, while still supporting bacterial escape from the phagocytic vacuole[26,27]; LLO^G486D^ does not cause early cell death in HUVEC (S1H Fig). To determine whether LLO^G486D^ supported invasion and vacuolar escape in HUVEC, we constructed an LLO^G486D^ strain (LLO^G486D^ *actAp*::*mTagRFP*, JAT983) that expressed RFP only when in the host cell cytoplasm[28]; we found that LLO^G486D^ mutants could invade HUVEC and escape the vacuole (Fig 1A).

In most cell types, *L*. *monocytogenes* replicates in the cytoplasm and expresses the protein ActA, which activates the Arp2/3 complex to promote actin polymerization at the surface of the bacterium[29,30]; addition of new actin subunits at the bacterial surface pushes the bacterium forward[31]. When a moving bacterium reaches the cell membrane, it can spread from cell to cell by extending a long membrane-bound protrusion that can be taken up by an adjacent cell into a double-membraned vacuole, from which the bacterium can again escape[12,19].

We found that LLO^G486D^ supported the ability of *L*. *monocytogenes* to move freely within cells (compare S1 and S2 Movies with *L*. *monocytogenes* expressing wild-type LLO in S3 Movie) and form bacterial protrusions that could extend from one endothelial cell and be internalized by an adjacent endothelial cell (S4 and S5 Movies).

**Fig 2:**
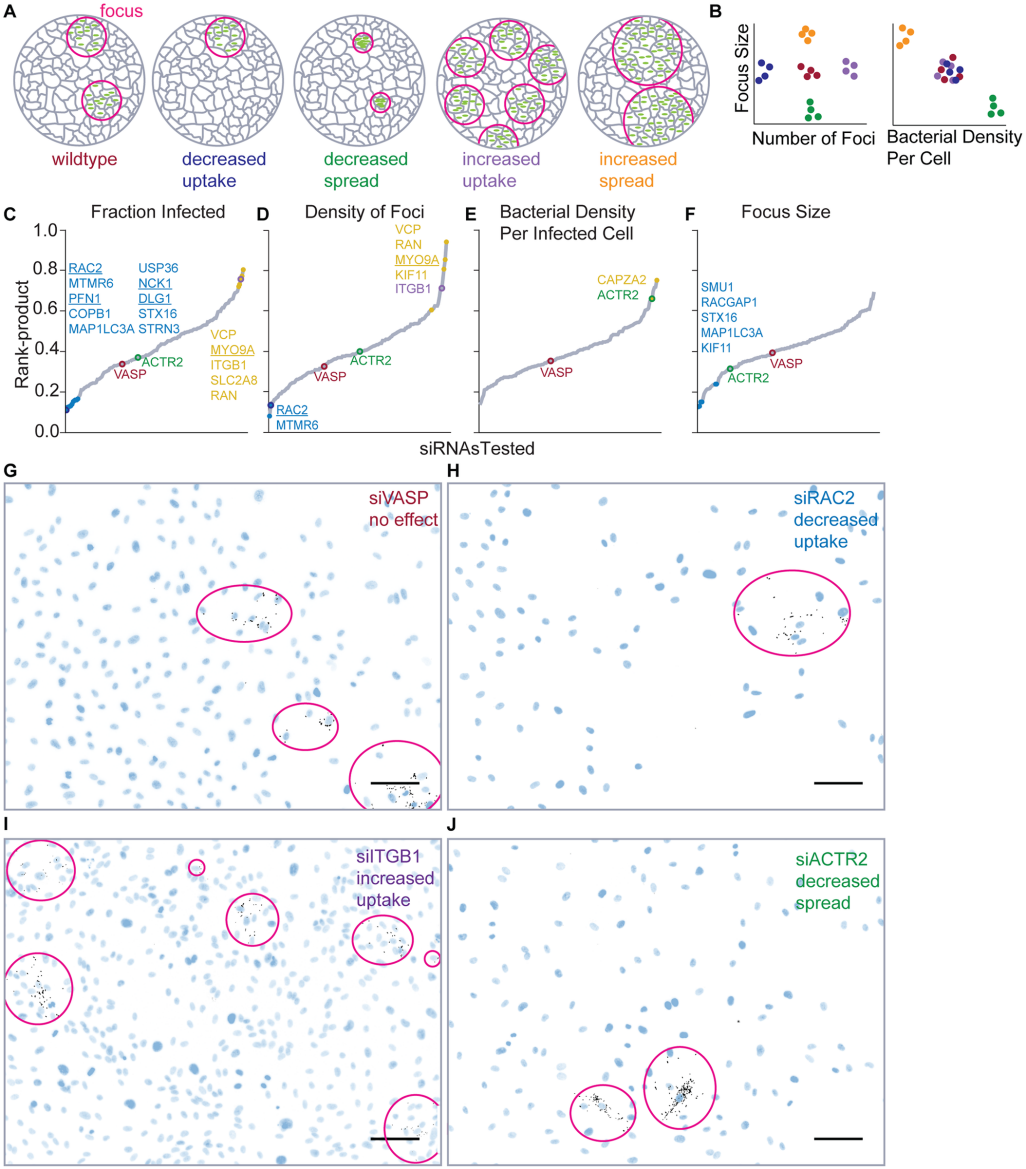
A quantitative image-based screen identified host factors that affect *L*. *monocytogenes* invasion and cell-to-cell spread. **(A**) Illustration of hypothetical phenotypes that can be identified in the screen. (**B**) Quantitative comparison of measurements in images of infected cells may decipher specific phenotypes. Colors indicate the phenotypes represented in (A). (**C-F**) Rank-product plots for quantitative metrics of invasion and spread. Gray line represents the distribution of rank-product values for the siRNAs, ordered by value. Statistically significant hits that are increased (yellow dots) or decreased (blue dots), relative to the average rank-product, are indicated and their names are listed in the corresponding color. siRNAs for which representative images are shown in G-J are highlighted and colored by phenotype. (**G-J**) Sample images from the screen exhibiting distinct phenotypes. Bacteria are shown in black and HUVEC nuclei are shown in blue. Foci are outlined. Scale bars: 50μm. (**G**) siVASP appeared in the center of the distribution for most metrics, indicating no significant phenotype. (**H**) siRAC2 exhibited few foci of normal size, indicating an invasion-specific defect. (**I**) siITGB1 exhibited many foci, indicating enhanced invasion. (**J**) siACTR2 exhibited foci with many bacteria per cell, indicating a cell-to-cell spread defect.

To confirm that LLO^G486D^ could propagate infection within an endothelial sheet, we employed the gentamicin protection assay, in which HUVEC were exposed to *L*. *monocytogenes* (JAT983) and, after 1 hour, the antibiotic gentamicin was added to specifically kill extracellular bacteria[21,32]; subsequent infection could occur only by cell-to-cell spread. We found an exponential increase in frequency of infected cells as a function of time (S2A, S2B and S2C Fig), indicating that *L*. *monocytogenes* expressing only LLO^G486D^ could indeed spread from an infected cell to an uninfected cell, escape from the secondary vacuole, and replicate in the newly infected cell. To quantify the extent of cell-to-cell spread, we evaluated the size of clusters of adjacent infected cells, termed foci. These foci represent an initial uptake event in a single cell, followed by subsequent cell-to-cell spread to neighboring uninfected cells (Fig 2A). The median focus remained stable for the first 6 hours of infection, then grew between 6 and 8 hours after infection, most likely representing the first successful cycle of cell-to cell-spread (S2D Fig). The significant motility of HUVEC in culture (S5 Movie) tended to fragment foci after 8 hours, so continuous spread was most evident by tracking the size of the largest decile of foci (S2D Fig). To quantify the contribution of cell-to-cell spread to overall infection of an endothelial sheet, we compared infection of the LLO^G486D^ mutant (JAT983) to an LLO^G486D^ *ΔactA* mutant (JAT985), which cannot polymerize actin and, therefore, cannot move within or between cells (S2E and S2F Fig). The number of foci, representing the number of distinct invasion events, was indistinguishable between JAT983 and JAT985 (S2H Fig), as expected, given that ActA is primarily expressed by intracellular bacteria and is not involved in invasion[29,33,34]. Compared to the ActA-deficient strain, JAT983 exhibited lower bacterial density in infected cells (S2I and S2J Fig) and larger focus size (S2K and S2L Fig), strongly suggesting that LLO^G486D^ supports cell-to-cell spread. Notably, these larger foci likely contribute to the higher percentage of cells infected with JAT983 versus JAT985 (S2G Fig). Collectively, these data demonstrate conclusively that LLO^G486D^ supported invasion, vacuolar escape, actin-based motility, and cell-to-cell spread in HUVEC without causing early cell death. We therefore used this mutant for all subsequent experiments in HUVEC.

Using the LLO^G486D^ mutant (JAT983), we determined that HUVEC are highly susceptible to *L*. *monocytogenes* invasion; at a high multiplicity of infection (MOI), more than 90% of HUVEC in a confluent monolayer harbored bacteria 8 hours after infection (Fig 1B). The amount of HUVEC infection is strongly dependent on MOI; thus, minor variations in MOI may result in substantially different frequencies of infected cells. Surprisingly, an LLO^G486D^ *inlAB* (JAT1119) mutant exhibited comparable invasiveness to JAT983 across the entire range of MOI examined; thus, neither of the canonical bacterial invasion proteins that promote uptake by intestinal epithelial cells and hepatocytes is required for internalization of *L*. *monocytogenes* into HUVEC (Fig 1B). Therefore, we suspected that either *L*. *monocytogenes* uses a different internalin-like protein to invade HUVEC or *L*. *monocytogenes* capitalizes on an intrinsic uptake mechanism in endothelial cells. To distinguish between these possibilities, we exposed HUVEC to *L*. *innocua*, which lacks most putative internalin family members and lacks all members with a known pathogenic role[23], or to polystyrene beads, which lack all bacterial factors. HUVEC were comparably susceptible to *L*. *monocytogenes* and *L*. *innocua* (Fig 1C and 1D). Surprisingly, HUVEC internalized polystyrene beads comparably to bacteria (Fig 1E and 1F). Concurrent exposure to *L*. *innocua* did not alter the frequency of HUVEC that internalized beads, suggesting that bacterial factors neither are required for nor enhance phagocytosis-like uptake by HUVEC (Fig 1F). Thus, *L*. *monocytogenes* likely exploits a generic constitutive uptake process in HUVEC without bacterial- or pathogen-specific requirements; such uptake may exhibit more similarity to a process like angiophagy or macrophage phagocytosis than to internalin-mediated invasion of epithelial cells[7,8].

### A quantitative image-based siRNA screen identifies factors that affect bacterial uptake and spread in primary human endothelial cells

To identify molecular regulators of endothelial phagocytosis-like uptake and *L*. *monocytogenes* infection, we performed a targeted siRNA screen, for which *in vitro* diced pools of siRNAs were generated, each targeting a distinct gene of interest (S1 Table) [35–37]. This method of generating complex siRNA pools, containing hundreds of different individual siRNAs, has been shown to reduce off-target effects often seen with single synthetic siRNAs by diluting the off-target effects of individual siRNAs in the pool[38]. We included genes that had previously been shown to modulate *L*. *monocytogenes* phagocytosis by macrophage-like *Drosophila* S2 cells[39,40] to compare that process to endothelial uptake. We also included components of cell-substrate adhesions and cell-cell contacts, as well as genes known to modulate collective motility, endocytic processes, intracellular trafficking, or membrane fusion.

Endothelial monolayers were infected with JAT983 in a gentamicin protection assay[21,32]. In normal infection, images of infected monolayers reveal multiple infection foci (Fig 2A and 2G). siRNA pools that specifically decrease uptake of bacteria should decrease the number of foci and the fraction of cells infected, but not focus size or the density of bacteria per infected cell (Fig 2A and 2H). Pools that specifically decrease cell-to-cell spread should decrease focus size while increasing the density of bacteria per infected cell, without changing the number of foci (Fig 2A and 2J). By combining multiple image-based metrics, distinct infection phenotypes may be extracted (Fig 2B). Notably, siRNAs could affect endothelial cell density, for instance by decreasing endothelial cell viability; if endothelial density affects *L*. *monocytogenes* internalization or spread, these siRNAs would have indirect effects on infection, but would be classified as significant in the screen. To correct our morphological metrics of infection for effects from changes in host cell density, we infected HUVEC that had been plated at varying densities and found that the frequency of infected cells (S3A Fig), bacterial density per infected cell (S3C Fig), and the size of the largest quartile of foci (S3D Fig) were uncorrelated with endothelial cell density. In contrast, the number of foci was linearly correlated with endothelial cell density (S3B Fig); therefore, we used the density of foci (number of foci divided by number of HUVEC) to quantify invasion independent of host cell density.

A number of siRNA pools caused phenotypes consistent with increased or decreased invasion (Fig 2C and 2D), while far fewer altered cell-to-cell spread (Fig 2E and 2F). To confirm that some siRNA pools specifically affected cell-to-cell spread, we examined the effect of 85 siRNAs from the original screen on infection of endothelial cells with an ActA-deficient strain (JAT1045) that is incapable of cell-to-cell spread; we included siRNAs that exhibited increased bacterial density per infected cell (siCAPZA2, siACTR2) or decreased focus size (siRACGAP1, siSTX16, siMAP1LC3A) in the original screen, expecting that these siRNA pools should not have a significant phenotype in this assay. We analyzed infection by flow cytometry (S4 Fig), which provided an orthogonal confirmation of the morphological metrics used in the initial screen. The candidates identified as likely to affect cell-to-cell spread in the original screen were not significantly different from controls in the *ΔactA* screen (S2 Table), confirming this interpretation. Notably, siITGB1 significantly increased infection with the ActA-deficient strain (S2 Table), consistent with its phenotype of increased bacterial invasion in the original screen (Fig 2C, 2D and 2I). Given that most of the candidate factors that appear to be involved only in cell-to-cell spread did not exhibit invasion phenotypes in this follow-up screen, we suspect that bacterial uptake and cell-to-cell spread are likely differentially regulated processes in endothelial cells.

### The formins *FHOD1* and *FMNL3* promote uptake of *L*. *monocytogenes* by endothelial cells

We were surprised to find that depletion of Arp2 yielded a phenotype consistent with a defect exclusively in cell-to-cell spread (Fig 2C, 2D, 2E and 2J and S2 Table), because previous studies have indicated that the Arp2/3 complex is the primary actin nucleator when *L*. *monocytogenes* invades epithelial cells and macrophages [40,41]. We confirmed this cell-to-cell spread-specific phenotype in HUVEC using synthetic siRNA pools that targeted distinct Arp2/3 subunits and successfully depleted the Arp complex (S5 Fig). Our phenotype was consistent with the known role of Arp2/3 in promoting *L*. *monocytogenes* actin-based motility and cell-to-cell spread [30] but demonstrated that bacterial uptake in HUVEC likely requires less Arp2/3 activity. Local actin polymerization is required by many cell types to internalize micron-sized objects, such as bacteria [42–44], and a subset of hits from the screen (underlined in Fig 2C and 2D), including *DLG1*[45], *NCK1*[46,47], *PFN1*[48], *RAC2*, and *MYO9A* [49,50], encode proteins that modulate actin assembly; depleting these proteins might alter the availability of cortical actin for local actin polymerization during bacterial internalization. Therefore, we examined whether actin polymerization during *L*. *monocytogenes* internalization by endothelial cells might be primarily controlled by the formin family of actin nucleators. Formin proteins contain multiple domains, including the formin homology-2 (FH2) domain, which binds to actin filaments and promotes elongation, and the FH1 domain, which modulates the activity of the FH2 domain by interacting with the actin monomer-binding protein profilin[48]. In our siRNA screen, depletion of profilin (*PFN1*) decreased the frequency of infected cells, consistent with an invasion defect (Fig 2C and S6A Fig).

**Fig 3:**
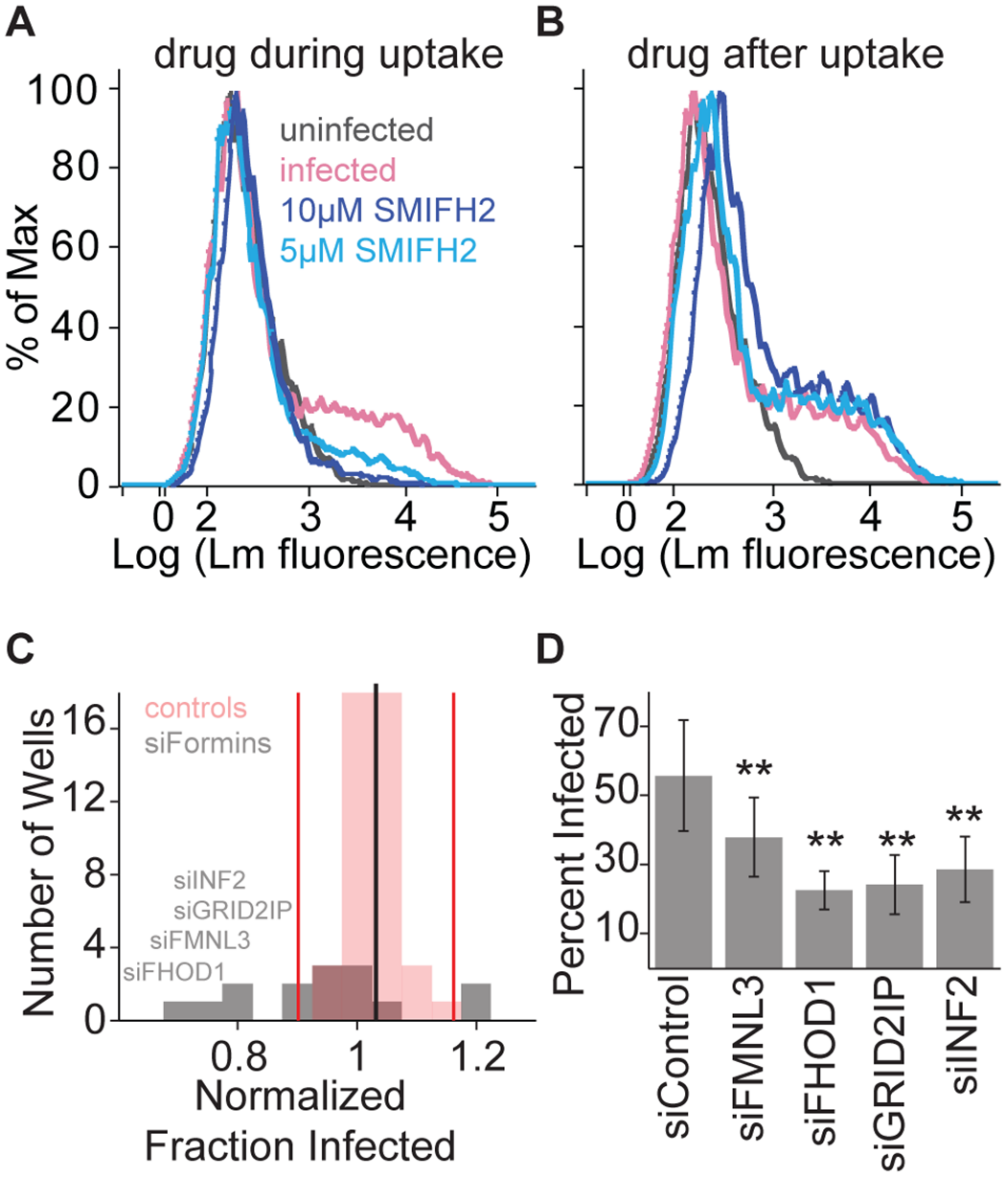
Formin activity is necessary for *L*. *monocytogenes* invasion in HUVEC. HUVEC were infected with JAT983. (**A, B**) Histograms of bacterial fluorescence intensity per cell. Infection was analyzed by flow cytometry 7–8 hours after infection. In each trace, a minor peak of higher bacterial fluorescence indicates infected HUVEC. (**A**) Effect of formin inhibition on bacterial uptake. SMIFH2 or vehicle control (DMSO) was present during invasion at the concentration indicated, and washed out with the addition of gentamicin. (**B**) Effect of formin inhibition on cell-to-cell spread. SMIFH2 or vehicle control was added with gentamicin and therefore was present only after invasion. (**C**) Effects of formin knockdown on bacterial uptake. HUVEC were treated with siRNAs targeting all 15 mammalian formins (gray bars) or non-targeting siRNAs (red bars) and analyzed by flow cytometry 7–8 hours after infection. The value for each sample is the average fold change (from 3 independent experiments with 4 biological replicates per experiment), relative to the mean percent infected among control siRNA wells (black line). Red vertical lines represent two SD from the mean. Names are listed for formins that were subsequently confirmed to be significantly different from controls. (**D**) Frequency of infected HUVEC (mean +/- SD, n = 4 biological replicates), for cells treated with siRNAs targeting *FMNL3*, *FHOD1*, *GRID2IP*, *INF2*, or non-targeting control siRNA, and analyzed by microscopy 8 hours after infection. P-values for each siRNA condition relative to control (unpaired two-sided, two-sample t-test, Benjamini-Hochberg correction): 0.0086 (siFMNL3), 1.0670 *10^−5^ (siFHOD1), 1.0670 * 10^−5^ (siGRID2IP), 0.0011 (siINF2).

A cell-permeable small molecule inhibitor of the FH2 domain (SMIFH2) broadly inhibits formin- but not Arp2/3-mediated actin polymerization[51]. We exposed HUVEC to the drug either during or after uptake of *L*. *monocytogenes* and assayed infection by flow cytometry (S4D Fig). HUVEC infection decreased when SMIFH2 was present during uptake (Fig 3A) but was comparable to the control when SMIFH2 was added after uptake (Fig 3B). These results suggest that formins normally promote uptake of *L*. *monocytogenes* by HUVEC but that their activity is not essential for cell-to-cell spread.

The human genome encodes 15 formins with distinct expression patterns, localizations, and functions[48,52]. Two of these formins, diaphanous-related formins 1 and 2 (*DIAPH1* and *DIAPH2*), were examined in our original screen, but did not exhibit a significant phenotype (S1 Table). To determine which formins were involved in uptake of *L*. *monocytogenes* by endothelial cells, we screened a targeted siRNA library that included each mammalian formin, and assayed infection by flow cytometry. We found that siRNAs targeting *FHOD1*, *FMNL3*, *GRID2IP* (Delphilin), or *INF2* exhibited significantly lower levels of *L*. *monocytogenes* infection than the control distribution (Fig 3C). To confirm our results, we also examined infection by microscopy after depletion of *FHOD1*, *FMNL3*, *GRID2IP* or *INF2*; all four decreased bacterial uptake, though depletion of *FMNL3* had the weakest effect (Fig 3D). By quantitative reverse transcriptase PCR, we reliably amplified *FHOD1*, *FMNL3*, and *INF2* in HUVEC, but did not detect expression of *GRID2IP*, and identical expression results have previously been reported for HUVEC and other endothelial primary cells [53] and in an endothelial-derived cell line (The Human Protein Atlas [54,55]). We suspect that this protein is not expressed in HUVEC and may not play a significant role in infection. We confirmed that the siRNAs targeting *FHOD1* and *FMNL3* reliably depleted their target mRNAs, however the siRNA targeting *INF2* minimally depleted *INF2* mRNA (S6B Fig). siRNAs targeting *INF2* and *GRID2IP* did not decrease levels of *FHOD1* or *FMNL3* mRNAs (S6C and S6D Fig), so their phenotype is most likely caused by other off-target effects. We therefore conclude that FHOD1 and FMNL3, and not Arp2/3, are the primary actin nucleators involved in internalization of *L*. *monocytogenes* by HUVEC.

### Focal adhesions inhibit uptake of *L*. *monocytogenes* by endothelial cells

FMNL3 and FHOD1 modulate actin dynamics in a number of critical cellular processes; in particular, both have been shown to interact with or modulate focal adhesions[56–58], large protein complexes that transduce mechanical and chemical signals between the cytoplasm and the extracellular matrix. Notably, the most robust invasion-specific hit in our screen came from siRNA pools targeting the focal adhesion protein integrin beta-1 (ITGB1), which increased the fraction of HUVEC infected and the density of foci in our original screen (Fig 2C, 2D and 2I) and also significantly increased infection of HUVEC with an ActA-deficient strain (S2 Table). Focal adhesions have not previously been implicated in *L*. *monocytogenes* invasion in non-phagocytic cell types, and depletion of focal adhesion proteins did not alter phagocytosis of *L*. *monocytogenes* by macrophage-like S2 cells[39,40].

**Fig 4:**
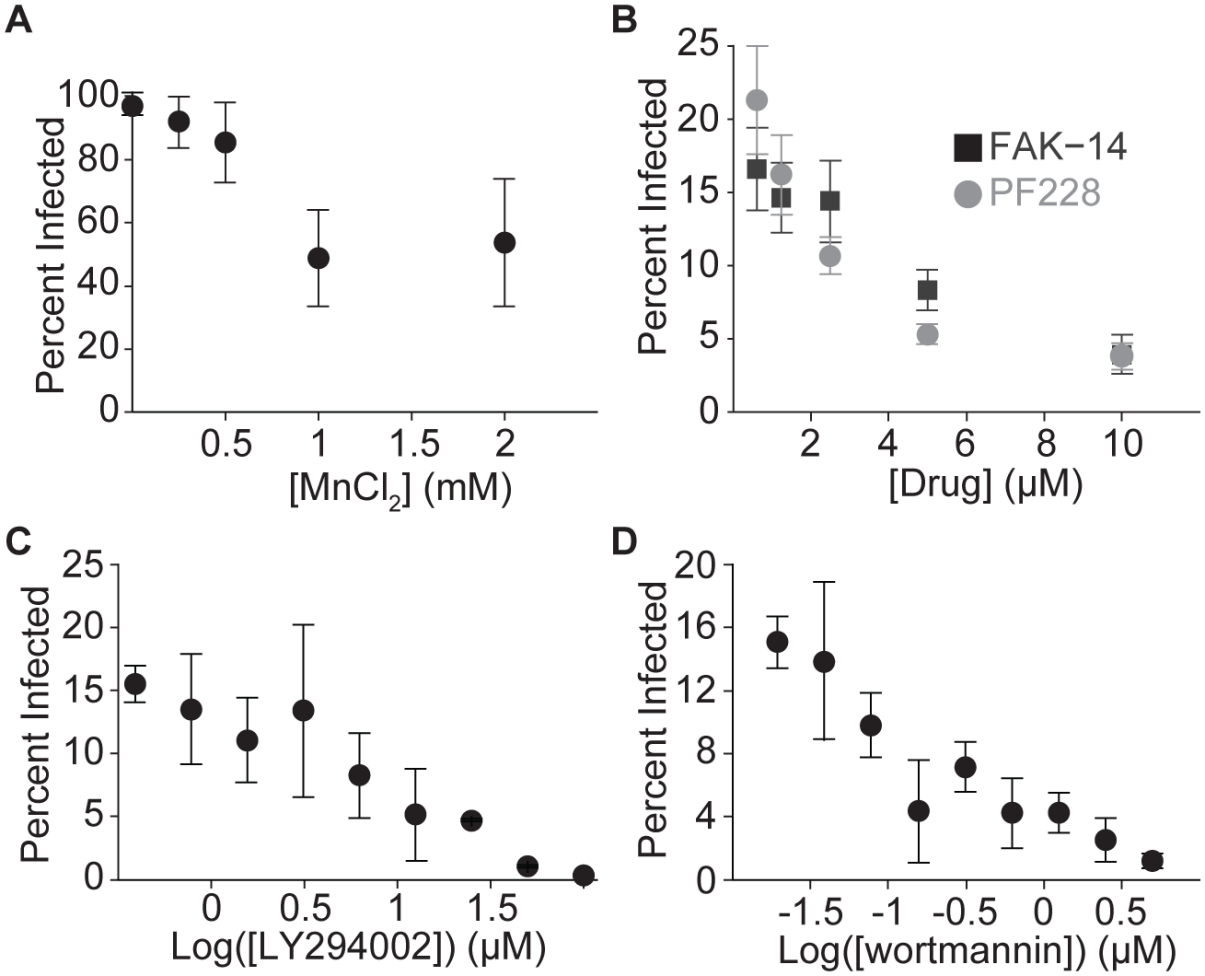
PI-3-kinase and focal adhesions modulate *L*. *monocytogenes* invasion in HUVEC. (**A-D**) Frequency of infected HUVEC as a function of inhibitor concentration (mean +/- SD, n = 4 biological replicates). Samples were infected with JAT1045. (**A**) Inhibition of bacterial uptake by manganese ions. MnCl_2_ or vehicle control (water) was added with bacteria. Infection was analyzed by microscopy 8 hours after infection. Representative data from 1 of 2 independent experiments. (**B**-**D**) Infection was analyzed by flow cytometry, 7–8 hours after infection. (**B**) Inhibition of bacterial uptake by FAK inhibitors. FAK-14 or PF573228 or vehicle control (DMSO) was added 40 minutes before addition of bacteria. Representative data from 1 of 3 independent experiments. (**C-D**) Inhibition of bacterial uptake by PI3K inhibitors. LY294002 or wortmannin or vehicle control (DMSO) was added 2 hours prior to addition of bacteria. Representative data from 1 of 2 independent experiments.

To complement siRNA experiments, which cause long-term depletion, we used small molecules to acutely perturb focal adhesions during bacterial uptake (S4D Fig). Furthermore, such pharmacological perturbations do not share the same off-target effects as siRNAs and, in particular, are independent from the entire process of RNA interference. Therefore, as with formins, use of both pharmacological and siRNA perturbations could provide independent confirmation of the role of focal adhesions in *L*. *monocytogenes* internalization by HUVEC. MnCl_2,_ which promotes the formation of focal adhesions by activating integrins[59], decreased uptake of *L*. *monocytogenes* (Fig 4A). Treating HUVEC with the focal adhesion kinase (FAK) inhibitors FAK-14 or PF573228 increased the frequency of abnormally large adhesions (S7 Fig), and therefore likely inhibited adhesion turnover. Both FAK inhibitors also inhibited uptake of *L*. *monocytogenes* in a dose-dependent manner (Fig 4B). The siRNA pools targeting FAK in our screen failed to deplete FAK mRNA (S6A Fig); thus it is not surprising that they did not exhibit a significant phenotype in the screen (S1 Table). Collectively, these data confirm that modulation of focal adhesions can inhibit uptake of *L*. *monocytogenes* by endothelial cells, as suggested by our siRNA screen (Fig 2C and 2D).

### A signaling pathway that regulates Rho activity via focal adhesions is necessary for uptake of *L*. *monocytogenes* by HUVEC

When endothelial cells are exposed to laminar shear (as might result from fluid in the bloodstream), a signaling pathway initiated at the apical surface promotes phosphoinositide 3-kinase (PI3K)-dependent reinforcement of focal adhesions, which signal through the small GTPase RhoA to increase cellular stiffness and cell-substrate adhesion[60–62]. PI3K has been shown to regulate *L*. *monocytogenes* invasion in other cell types[63], and our data demonstrate a clear role for FAK in promoting *L*. *monocytogenes* uptake in HUVEC. Therefore, we wondered if other elements of the shear-stress responsive pathway might be involved as well. Indeed, the PI3K inhibitors LY294002 and wortmannin both decreased uptake of *L*. *monocytogenes* in a dose-dependent manner (Fig 4C and 4D).

RhoA activity is decreased by GTPase activating proteins (GAPs), which promote GTP hydrolysis, and is increased by guanine nucleotide exchange factors (GEFs), which promote the exchange of GDP for GTP[64]. In our screen, the siRNA pool targeting the RhoGAP MyosinIX (*MYO9A*) increased the fraction of cells infected and the density of foci (Fig 2C and 2D). While RhoA has been implicated in *L*. *monocytogenes* invasion of epithelial cells[65], depletion of RhoA increased uptake of *L*. *monocytogenes* by S2 cells[40], exactly the opposite of the result suggested by siMyo9A in our screen in HUVEC.

**Fig 5:**
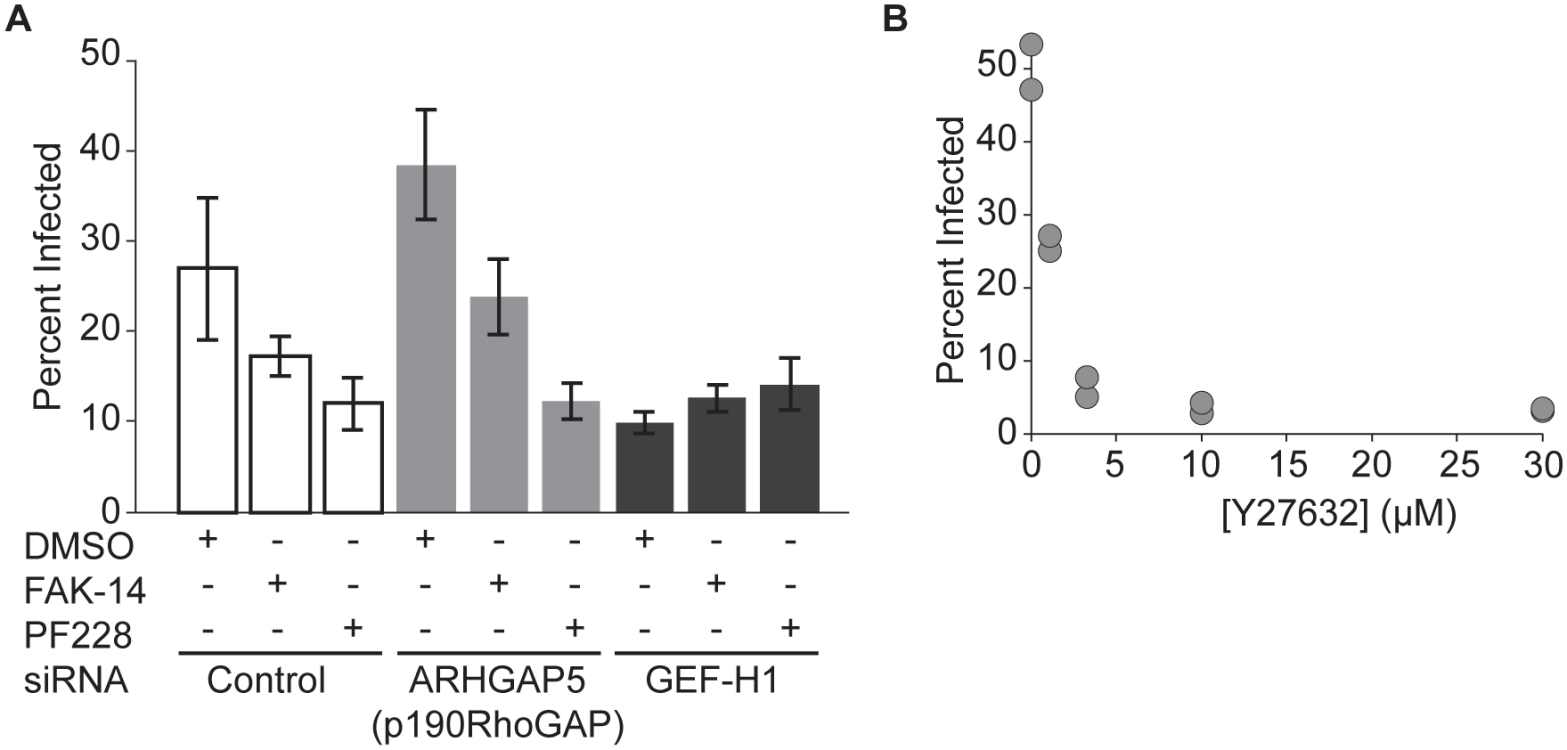
FAK regulates uptake of bacteria by modulating the activity of Rho and Rho kinase. (**A**) Effect of FAK inhibition after siRNA treatment targeting *ARHGAP5* (p190RhoGAP), ARHGEF2 (GEF-H1) or non-targeting siRNA controls. DMSO (vehicle control), 10μM FAK-14, or 10μM PF573228 was added 40 minutes prior to infection with JAT983. Infection was analyzed by microscopy (mean +/- SD, n = 4 biological replicates) 8 hours after infection. (**B**) Frequency of infected HUVEC as a function of Y27632 concentration (n = 2 biological replicates). Y27632 was added 30 minutes prior to infection with JAT1045; infection was analyzed by flow cytometry 7–8 hours after infection. Representative data from 1 of 2 independent experiments.

Nascent focal adhesions inhibit Rho activity via p190RhoGAP (*ARHGAP5*)[66]; however, FAK can also promote Rho activity via the RhoGEF GEF-H1[61,62]. To distinguish between these pathways, we exposed cells to siRNAs targeting p190RhoGAP or GEF-H1. When p190RhoGAP was depleted, *L*. *monocytogenes* internalization by HUVEC was still decreased by FAK inhibition (Fig 5A). In contrast, depleting GEF-H1 (S6B Fig) reduced the frequency of infected HUVEC, and FAK inhibition did not affect uptake of *L*. *monocytogenes* when GEF-H1 was depleted (Fig 5A), indicating that GEF-H1 acts downstream of FAK in this pathway. Thus, we concluded that FAK signaling normally increases Rho activity via GEF-H1 to promote uptake of *L*. *monocytogenes*.

When we acutely treated cells with Y27632[67], which inhibits the major Rho effector, Rho kinase (ROCK), the frequency of infected cells decreased in a dose-dependent fashion (Fig 5B), indicating that ROCK activation is necessary during uptake of bacteria. As with FAK, the siRNA pools in the original screen did not exhibit a significant phenotype (S1 Table), but also only moderately depleted ROCK mRNA (S6A Fig). Furthermore, an acute perturbation in ROCK activity may be more indicative of a specific role in bacterial uptake than long-term depletion, which may be accompanied by other cytoskeletal remodeling.

The siRNA pool targeting RhoA effectively depleted its target mRNA (S4A Fig) but did not exhibit a significant phenotype in the screen (S1 Table). Redundant function of RhoA, B, and C may contribute to the lack of phenotype when only RhoA is depleted [68]. It is also probable that RhoA has multifaceted effects on *L*. *monocytogenes* infection; it could influence both global and local actin dynamics, which might have opposing effects on *L*. *monocytogenes* internalization.

### ROCK and formins distinctly regulate bacterial adhesion and endothelial phagocytosis-like uptake, respectively

**Fig 6:**
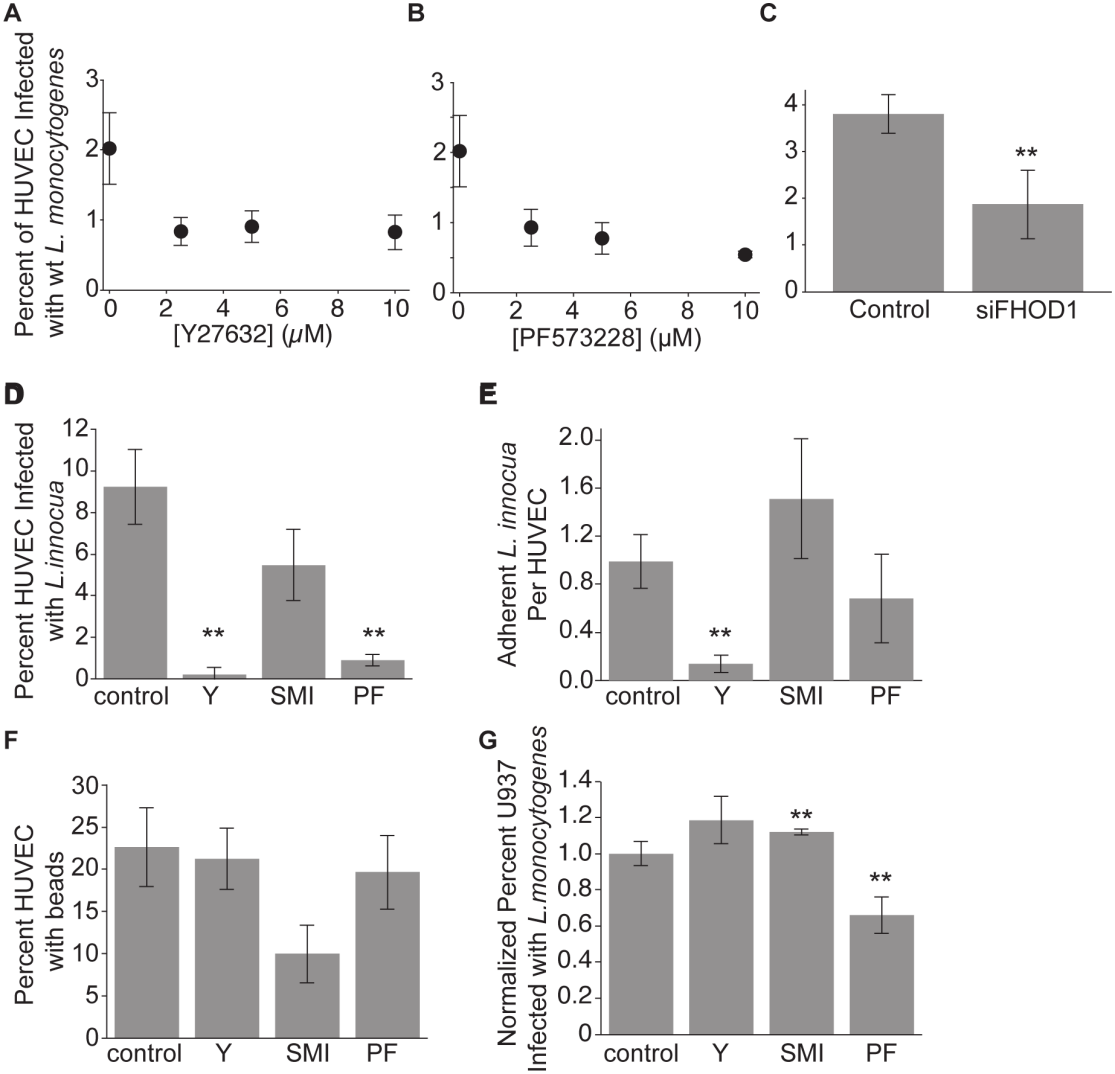
ROCK and formins modulate different steps of the bacterial internalization process in HUVEC and do not affect macrophage phagocytosis of *L*. *monocytogenes*. DMSO (vehicle control), 10μM Y27632, 10μM SMIFH2, or 10μM PF573228 were added 40 minutes prior to infection. (**A**-**C**) HUVEC were infected with low doses (MOI < 10) of *L*. *monocytogenes* (JAT607) expressing wild-type LLO and analyzed by flow cytometry 7–8 hours after infection (mean +/- SD, n = 4 (**A**, **B**) or 8 (**C**) biological replicates). (**A**) Inhibition of HUVEC uptake of wild-type *L*. *monocytogenes* by Y27632. (**B**) Inhibition of HUVEC uptake of wild-type *L*. *monocytogenes* by the FAK inhibitor PF573228. (**C**) Inhibition of HUVEC uptake of wild-type *L*. *monocytogenes* by knockdown of FHOD1. HUVEC were treated with siRNAs targeting FHOD1 or non-targeting controls. P-value (unpaired two-sided t-test) = 0.0037. **(D-F**) HUVEC were exposed to *L*. *innocua* or beads and analyzed by inside/outside staining as in Fig 1. ** indicates significance at p<0.05 (**D**) Effects of inhibitors on uptake of *L*. *innocua*. Frequency of infected HUVEC (mean +/- SD for n = 3 biological replicates). P-values for each drug treatment relative to control (unpaired two-sided, two-sample t-test, Benjamini-Hochberg correction): 0.0256 (Y27632), 0.0735 (SMIFH2), 0.0266 (PF573228). (**E**) Effect of inhibitors on bacterial adhesion. Average number of adherent *L*. *innocua* per HUVEC. P-values for each drug treatment relative to control (unpaired two-sided, two-sample t-test, Benjamini-Hochberg correction): 0.0094 (Y27632), 0.2577 (SMIFH2), 0.2853 (PF573228). (**F**) Effect of inhibitors on bead uptake. Frequency of HUVEC with internalized beads (mean +/- SD for n = 3 biological replicates). P-values for each drug treatment relative to control (unpaired two-sided, two-sample t-test, Benjamini-Hochberg correction): 0.4664 (Y27632), 0.0583 (SMIFH2), 0.4664 (PF573228). (**G**) Normalized frequency of infected U937 (mean +/- SD for n = 4 biological replicates from 2 independent experiments). U937 cells were infected with *actAp*::*RFP L*. *monocytogenes* (JAT607), and analyzed by flow cytometry. P-values for each drug treatment relative to control (Wilcoxon rank sum test, Benjamini-Hochberg correction), for each experiment: 0.0857 (Y27632), 0.0429 (SMIFH2), 0.0429 (PF573228).

Our data indicate that FAK- and GEF-H1-dependent ROCK activity and also formin-mediated actin polymerization promote uptake of *L*. *monocytogenes* by HUVEC; however these data were all obtained using strains with the LLO^G486D^ point mutation, given the substantial susceptibility of HUVEC to LLO. To verify that the presence of wild-type LLO would not significantly change the process of internalization, we examined the effects of pharmacological FAK and ROCK inhibition and FHOD1 depletion during very low dose infection with wild-type *L*. *monocytogenes* (JAT607) and found that internalization of wild-type *L*. *monocytogenes* by HUVEC is strongly FAK-, ROCK-, and FHOD1-dependent (Fig 6A, 6B and 6C).

If *L*. *monocytogenes* capitalizes on an intrinsic, constitutive, phagocytosis-like process, then this same signaling pathway should be necessary for uptake of other large objects by HUVEC. First, we examined whether inhibition of ROCK or FAK could substantially reduce the ability of HUVEC to internalize *L*. *innocua* as well as *L*. *monocytogenes* and found this to be true (Fig 6D). Similarly, formin inhibition reliably reduced internalization of *L*. *innocua* (Fig 6D).

Phagocytosis-like uptake could be modulated by changes in bacterial adhesion to cells, in the internalization process itself, or in changes in global cellular state, such as cell stiffness, that might indirectly affect adhesion or internalization. To differentiate between these possibilities, we quantified adhesion of *L*. *innocua* in the presence of ROCK, FAK, or formin inhibitors and found, surprisingly, that ROCK inhibition, but not inhibition of formins or FAK, dramatically reduced *L*. *innocua* adhesion to HUVEC (Fig 6E). Furthermore, inhibition of ROCK did not affect the ability of HUVEC to internalize beads, which adhere non-specifically (Fig 6F), consistent with a role for ROCK specifically in bacterial adhesion, rather than internalization. Formin inhibition did decrease internalization of beads by about 50% (Fig 6F), comparable to its effect on *L*. *innocua* internalization (Fig 6D) and to the effect of siFHOD1 and siFMNL3 on *L*. *monocytogenes* internalization (Fig 3D); however, formin inhibition did not inhibit bacterial adhesion (Fig 6E). Therefore, we conclude that formins are involved in actin remodeling specifically during phagocytosis-like uptake. Surprisingly, FAK inhibition decreased *L*. *innocua* and *L*. *monocytogenes* internalization without affecting *L*. *innocua* adhesion, but had no effect on internalization of beads (Fig 6F).

Finally, we examined whether these regulators of bacterial uptake by HUVEC affected macrophage phagocytosis of *L*. *monocytogenes*. Only inhibition of FAK disrupted phagocytosis of *L*. *monocytogenes* by activated U937 cells, a macrophage-like cell line, whereas ROCK and formin inhibition had no significant effect (Fig 6G). Furthermore, it has previously been shown that ROCK inhibition does not affect uptake of *L*. *monocytogenes* by a variety of macrophage-like cell lines[69]. Therefore, we conclude that endothelial cells and macrophages use distinct pathways to internalize *L*. *monocytogenes*.

## Discussion

### Cytoskeletal regulation of bacterial internalization by endothelial cells is multi-faceted

Our data demonstrate that a number of proteins in the endothelial shear stress-responsive pathway, including PI3K, FAK and focal adhesions, GEFH1, and ROCK, also regulate *L*. *monocytogenes* internalization in endothelial cells; however, it is also clear from our data that the PI3K/FAK/RhoA pathway is not activated in a straightforward linear manner that starts with PI3K activity and culminates in bacterial uptake. For instance, although our data demonstrate that FAK activity is upstream of GEFH1, implying that it is upstream of ROCK, FAK inhibition inhibits bacterial uptake but not adhesion, whereas ROCK inhibition dramatically inhibits bacterial adhesion. Likewise, although ROCK has previously been shown to directly phosphorylate and activate FHOD1[70], formin inhibition did not affect bacterial adhesion in our assay, and formin inhibition, but not ROCK inhibition, inhibited phagocytosis-like internalization of polystyrene beads. The PI3K/FAK/Rho signaling pathway normally promotes global rearrangements in endothelial cell architecture in response to apical signaling events and mechanical deformation[62], however many of these proteins can also act locally to modulate the chemical and mechanical environment; these local and global effects may even have opposing effects on bacterial internalization or phagocytosis-like uptake of other large objects. Here, we have identified specific proteins required for bacterial adhesion to and internalization by endothelial cells; further experiments that simultaneously combine both precise spatial and temporal control of protein activity will be necessary to dissect multiple global and local roles of these proteins during infection.

We have shown that *L*. *monocytogenes* and *L*. *innocua*, a non-pathogenic relative, are internalized at comparable rates and are regulated by similar host effectors; therefore, no *Listeria monocytogenes*-specific effectors were necessary for bacterial internalization in HUVEC. Additionally, we have shown that microspheres are internalized at least as efficiently as bacteria, and that the presence of *L*. *innocua* did not further enhance microsphere uptake. Therefore, HUVEC exhibit constitutive phagocytic behavior that is not enhanced or inhibited by the presence of bacteria. Notably, we also show that ROCK-independent adhesion of beads appears to be distinct from ROCK-dependent adhesion of bacteria, although both uptake processes are formin-dependent to a comparable degree. Thus, while bacterial and bead adhesion are differentially regulated, our results suggest that, once adhered, the internalization mechanism is similar and formin-dependent.

### *L*. *monocytogenes* uses distinct modes of invasion in distinct host cell types

A hallmark of systemic listeriosis is the ability of *L*. *monocytogenes* to infect distinct cell types in distinct organs, including intestinal epithelial cells, hepatocytes, placental cytotrophoblasts, endothelial cells, macrophages and other immune cells[5]; *L*. *monocytogenes* invades these distinct cell types using both pathogen-triggered and pathogen-independent mechanisms. Intriguingly, *L*. *monocytogenes* uses distinct invasion mechanisms that capitalize on unique characteristics of these different cell types. For instance, *L*. *monocytogenes* uses the epithelial junctional protein E-cadherin to invade intestinal epithelial cells[7,8] and the hepatocyte growth factor receptor c-Met to invade hepatocytes[9,10]; such invasion requires the *L*. *monocytogenes* proteins InlA and InlB, respectively. In contrast, in this context, endothelial cells may behave more like professional phagocytes, with internalization less likely to be pathogen-specific and more likely to involve *Listeria* adhesion followed by co-option of a normal constitutive phagocytosis-like uptake process. Notably, the endothelial factors involved are critical endothelial regulators, including the PI3K/FAK/Rho shear stress-signaling pathway and FMNL3, an endothelial formin that is critical for angiogenesis[53,71,72]. Although macrophages have been shown to use formins and ROCK [73,74] during phagocytosis of some cargo, we found that inhibition of these proteins did not inhibit macrophage-like cells from internalizing *L*. *monocytogenes*; thus, *L*. *monocytogenes* hijacks unique and distinct pathways in macrophage and endothelial infection. Given dramatically different kinetics of macrophage phagocytosis and endothelial phagocytosis-like uptake *in vivo*[1,75], and that the PI3K/FAK/Rho signaling axis regulates endothelial architectural changes, we speculate that substantial remodeling of the endothelial cytoskeleton is required for phagocytosis-like uptake and may explain its slower kinetics.

### Endothelial phagocytosis-like uptake may be a bloodstream surveillance strategy

Although endothelial phagocytosis-like uptake occurs in a number of different contexts *in vivo* and in culture, its role *in vivo* is unclear. Here, we determined that the formins FMNL3 and FHOD1 likely regulate such uptake; genetic and pharmacological perturbation of these proteins can now be used to understand the role of phagocytosis-like uptake *in vivo*.

We speculate that endothelial phagocytosis-like uptake is a surveillance strategy to remove particles from the bloodstream, particularly in cases of macrophage injury, or at sites at which macrophages have limited access. For instance, angiophagy might enhance fibrin clot clearance and restore blood flow in small diameter vessels that do not receive much immune cell traffic[1,2].

Phagocytosis-like uptake by endothelial cells may also recruit immune cells specifically to vulnerable sites in the vasculature to limit pathogen dissemination. Indeed, endothelial cells increase expression of pro-inflammatory cytokines and chemokines in response to *L*. *monocytogenes*[76] and *Rickettssiae*[77], which comprise a group of obligate intracellular bacterial species that cause spotted fever and typhus and preferentially infect endothelial cells, likely through a direct receptor-mediated process[78,79]. Additionally, endothelial cells have been shown to kill internalized *Rickettssiae* directly in a cytokine-activated hydrogen peroxide- or nitric oxide-dependent manner[77,80], and thus may contribute directly to pathogen removal.

In such a setting, escape from the vacuole may be the primary pathogenic strategy of intracellular bacteria. Indeed, like *L*. *monocytogenes*, *Rickettssiae* species can escape the vacuole and proliferate in the endothelial cell cytoplasm[81,82]. Furthermore, both *L*. *monocytogenes*[12,19,31] and *Rickettssiae* species[81,82] can hijack host cell actin to move within and between cells, without exposure to the extracellular space. Thus, these pathogens may re-direct their own dissemination, rather than passively following transcytosis; this may be a mode of *L*. *monocytogenes* spread across the endothelium into the central nervous system.

## Materials and Methods

### Bacterial strain construction

S3 Table lists bacterial strains used in this study. To express fluorescent proteins in *L*. *monocytogenes* strains, plasmids were transformed into *E*. *coli* SM10 **λ**pir by electroporation and subsequently transferred to *L*. *monocytogenes* by conjugation[83]. Constructs were stably integrated into the tRNA^ARG^ locus of the bacterial chromosome as previously described[83]. For constitutive GFP expression, plasmid pMP74 (a gift from M. Pentecost and M. Amieva), in which sGFP is expressed under the Hyper-SPO1 promoter fused to the 5′ UTR of *hly*[84,85], was incorporated into JAT745 or JAT984 to generate strains JAT1045 and JAT1046, respectively. An identical approach was used to express a codon-optimized mTagRFP under the control of the ActA promoter (plasmid pPL499[28], a gift from P. Lauer) to generate strains JAT983 and JAT985, respectively, which express mRFP only after reaching the host cell cytoplasm.

The *inlAB* LLO^G486D^ strain was generated by integrating the LLO^G486D^ mutation into JAT1084 (a gift from M. Pentecost and M. Amieva), by allelic exchange[26,86,87] to generate JAT1116. Integration was verified by sequencing the *hly* locus. Codon-optimized mTagRFP (from pPL499) was incorporated into JAT1116 as described above to generate strain JAT1119.

### Mammalian cell growth conditions

HUVEC (Lonza C2517A) were cultured according to the manufacturer’s instructions (EGM Bullet Kit-2, Lonza CC-3162). Infections were performed in normal growth media but lacking serum and antibiotics (serum- and antibiotic-free media, SAFM). For microscopy experiments, black 96-well clear-bottom plates (E&K Biosciences 25090) or glass coverslips were coated with 30μg/mL collagen type I in PBS (Advanced Biomatrix 5005-100ML) for 1 hour at 37°C and then washed once with PBS before cells were plated. U937 cells (ATCC, CRL-1593.2) were grown in RPMI with 10% fetal bovine serum and gentamicin/amphotericin (Lonza, CC-4083); for these cells SAFM consisted of RPMI without additives.

### Antibodies and reagents

DAPI (Invitrogen D1306) was dissolved at 5mg/mL in dimethyl formamide and used at 1/5000. Other drugs were dissolved in DMSO (endotoxin-free dimethyl sulfoxide, Sigma D2650) at stock concentrations indicated below. Stock concentrations and sources of drugs were: 50mM LY294002 (Sigma L9908), 25mM wortmannin (EMD Chemicals 12–338), 30mM Y27632 (EMD Chemicals 688000), 10mM FAK inhibitor-14 (FAK-14) (Tocris Bioscience 3414), 100mM PF573228 (Tocris Bioscience 3239). SMIFH2 (Millipore 344092) solutions were freshly made with each experiment as we found that frozen stocks degraded over time. Primary antibody used for inside/outside staining was BacTrace anti-*Listeria* genus specific antibody (01-90-90, KPL, Inc.). Fluorescent streptavidins used for inside/outside staining of 2.0μm biotinylated polystyrene beads (Polysciences, Inc. 24172) were Alexa-Fluor-546-streptavidin (Invitrogen S11225) and Alexa-Fluor-488-streptavidin (Invitrogen S11223). For Western blotting, rabbit monoclonal anti-Arp2 antibody (Epitomics 5738–1) was used to detect Arp2.

### Endothelial cell infection

Endothelial cells were infected as previously described[21,32] with the following modifications. *L*. *monocytogenes* liquid cultures were started from a plate colony, and grown overnight, spinning, at 30°C in Brain Heart Infusion (BHI) media (Gibco 211059) supplemented with 200μg/mL streptomycin. Chloramphenicol-resistant strains were grown with 7.5 μg/mL chloramphenicol. Cultures were diluted in fresh media to an OD600 of 0.1 and returned to a spinning wheel at 30°C for 2–2.5 hours. Bacteria were then washed 3 times with PBS to remove any soluble factors and diluted into SAFM. HUVEC were washed once with SAFM, and bacteria were added to an MOI of 50–100 bacteria per HUVEC unless otherwise indicated. For every experiment, MOI was calculated directly by counting the colony forming units in the bacterial inoculum. To synchronize invasion, samples were spun for 10 minutes at room temperature at 500 x g prior to incubation. After thirty minutes, samples were washed four times with SAFM and, after an additional thirty minutes, media was replaced with SAFM supplemented with 20μg/mL gentamicin. Analysis was performed by flow cytometry or microscopy at 8 hours after exposure, unless otherwise indicated.

Samples analyzed by microscopy were fixed for fifteen minutes in 3.7% formaldehyde buffered in sodium phosphate, stained with DAPI, and imaged on an ImageXpress Micro (Molecular Devices) using a 10X or 20X air objective; the percent of cells infected was determined as described below for the siRNA screen. For each biological replicate, 300–500 cells were analyzed. Analysis of experiments in Figs 1D, 1F and 6 was manual but the experimenter was blinded to the identity of samples during imaging and analysis. Analysis of experiments in Fig 1B and Figs 2–5 was automated as described below in *Analysis of the siRNA Screen*.

For drug experiments (Figs 3–6), unless otherwise indicated, media was removed from cells and replaced with media containing either the drug or DMSO (vehicle control), either at the time of infection or prior to infection. Cells remained in media containing the drug until 1 hour after infection, when cells were washed twice and replaced with drug-free gentamicin-containing media.

### LLO sensitivity experiments and flow cytometry

HUVEC or U937 were exposed to bacteria or 6-His-LLO for 30 minutes. HUVEC were washed in PBS and then incubated in 0.25% trypsin-EDTA (Invitrogen) for 15–20 minutes to fully detach all cells; an equal amount of 6% fetal bovine serum in PBS was then added to inactivate the trypsin. U937 were in solution throughout the experiment. Propidium iodide was added to cells in solution at a final concentration of 25μM and samples were immediately analyzed on a BD LSRII Flow Cytometer (BD Biosciences). Live cells were identified as described in S1A, S1B and S1C Fig For infection experiments, we determined the fraction of HUVEC that were infected as illustrated in S4 Fig For each biological replicate, 5,000–10,000 cells were analyzed. His-LLO was purified as described[24] and provided by Jennifer Robbins and Lisa Cameron.

### Bacterial or bead adhesion assays and inside/outside staining

Samples were infected as described above and fixed 30 minutes after initial exposure to bacteria or beads. To quantify bacterial adhesion and internalization, inside/outside staining was performed as described previously[88], using the BacTrace anti-*Listeria* genus primary antibody or fluorescent streptavidin conjugates. Samples were additionally stained with DAPI to identify HUVEC nuclei. Coverslips were mounted onto slides with VectaMount (Vector Labs). Samples were imaged on a Nikon Eclipse TiE inverted fluorescence microscope equipped with a charge-coupled device (CCD) camera (Andor Technologies) using a 63X or 100X oil objective, and captured with the Micromanager[89] software package. HUVEC were identified from transmitted light images and DAPI staining. All bacteria or beads associated with individual HUVEC were counted as adherent; bacteria or beads that lacked the “outside” stain (applied before permeabilization) were counted as internalized.

### Diced siRNA library construction and endothelial cell transfection

To minimize off-target effects and maximize on-target effects, siRNA pools targeting candidate genes of interest were produced by *in vitro* dicing as previously described[35,36] using purified *Giardia* Dicer[37]. Due to low yield for some pools in our first synthesis, we performed the synthesis twice to include all of our candidates. To avoid positional effects, the position of each siRNA pool in the final 96-well plates was randomized.

For each well of a 96-well plate, 10^4^ HUVEC suspended in SAFM were reverse-transfected with siRNAs at 20 nM final concentration using 0.25 μL Lipofectamine RNAiMAX (Invitrogen 13778075). The transfection mix was replaced by SAFM 8–9 hours later. Synthetic siRNAs for targeting genes of interest (Figs 3 and 5) were purchased from Dharmacon (S4 Table). Infections were performed approximately 72 hours after transfection.

### Analysis of siRNA screen

We screened each siRNA pool in 6 replicates on 3 different days for each of the 2 independent siRNA syntheses; thus, for most candidates, we collected data from 12 independent replicates. To correct for day-to-day variability in the infection itself, each plate included 10 wells of HUVEC that were not treated with siRNA and were exposed to either JAT983 or JAT985. siRNA-treated wells were infected with JAT983 at an MOI of 50–100.

For each image, Cell Profiler[90] was used to identify nuclei and to estimate cell boundaries. Infected cells were defined using a background threshold on the images of bacteria. Foci were defined as groups of contiguous infected cells. Bacterial density in an infected cell was defined as the number of pixels in the cell above the threshold that defined the signal from bacterial fluorescence. Foci consisting of a single, unreplicated bacterium in a single cell were removed from analysis; such filtering maximized the difference between JAT983- and JAT985-infected samples. For each siRNA, in each replicate, we quantified: the fraction of HUVEC infected, the top quartile of bacterial density per infected cell, the density of foci, the top quartile of focus size, and the number of HUVEC (used to calculate density of foci). We used the top quartile rather than median for the bacterial density and focus size measurements because these maximized the difference between JAT983- and JAT985-infected wells.

To identify specific outliers, we used the rank-product for each metric, which corresponds to the geometric mean of the rank of each siRNA pool in each experiment, and has been used to determine outliers from microarray data[91]. Briefly, siRNA-treated wells in each replicate were ordered and assigned the rank of p/n, where p is the well’s position in the ordered list and n is the total number of siRNA-treated wells in that replicate. The rank-product for all the replicates of a given siRNA is then given by (Π_i_^r^p_i_/n_i_)^(1/r), where r is the total number of replicates of that siRNA, p_i_ is the ranking in the ith replicate, and n_i_ is the number of RNAs in the ith replicate[91]. If all siRNA pools had the exact same effect, then each one would have a ranking that converged to 0.5 with increasing experimental replicates.

To generate the null distribution (for which we assume that all siRNAs gave identical effects), we performed identical analysis except that the names of siRNAs were randomly permutated prior to calculating the rank-product; we ranked 20,000 such permutation simulations to capture the probability of relatively rare events. To identify the statistical outliers in our data, we calculated the frequency of a particular siRNA’s rank among the simulations. To correct for multiple hypothesis testing (since we screened 156 individual RNAs), we used the Benjamini-Hochberg Procedure to hold the false discovery rate to 0.05.

### RT-qPCR and western blotting

HUVEC were treated with control or experimental siRNA as described above. mRNA was harvested using the RNeasy Micro Kit (Qiagen 74004) and cDNA was prepared using the Superscript III First-strand Synthesis SuperMix (ThermoFisher 18080–400). Genes of interest were amplified using primers specified in S5 Table. qPCR was performed using SYBR Select Master Mix (ThermoFisher 4472908) on a StepOnePlus Real-Time PCR System. Normalized relative quantity (NRQ) and error were calculated as previously described[92]. CDH5, ACTR2, MYH9, and GAPDH were used as control genes.

For Western blotting, samples were treated with siRNAs as described above. After 72 hours of depletion, cells were lysed in SDS sample buffer (2% SDS, 10% glycerol, 0.02% bromophenyl blue sodium salt, 1% beta-mercaptoethanol, 5mM EDTA, 80mM Tris-HCl pH6.8), sonicated and boiled for 10 minutes each. Samples were run on 12% SDS-PAGE gels, transferred to nitrocellulose membrane via semi-dry transfer. Total protein was evaluated by staining in Ponceau-S (0.2% Ponceau-S, 3% trichloroacetic acid, 3% sulfosalicylic acid). Membranes were then blocked in milk and stained with anti-Arp2 primary antibody, then horseradish peroxidase-conjugated goat anti-rabbit secondary antibody, and visualized by chemiluminescence.

### U937 infections

U937 cells were differentiated with phorbol 12-myristate 13-acetate (PMA) at 80nM for 36–48 hours prior to infection and were noted to be adherent at the time of infection. Infections were performed exactly as described above for endothelial cells except that U937 were infected with ActA-deficient *L*. *monocytogenes* expressing wild-type LLO (JAT610), and adherent U937 cells were infected directly from overnight liquid culture at an MOI of 80. Infection was analysed 7 hours after infection by flow cytometry as previously described.

## Supporting Information

S1 Fig HUVEC are highly sensitive to listeriolysin O. (**A-C**) Quantification of healthy cells by flow cytometry. (**A**) Single cells isolated by forward scatter area and side scatter area. The bulk of the distribution in the forward scatter area vs. side scatter area plot (enclosed by the red “scatter” gate) is single cells. (**B**) Refinement of single cell population using forward scatter height. The contents of the scatter gate (in A) are again gated to collect the bulk of the distribution on the forward scatter area vs. height plot. Outliers are more likely to be doublets or triplets. (**C**) Isolation of live cells. The contents of the single cell gate (in B) are gated to collect the live cells, which have not taken up propidium iodide. (**D-G**) Number of healthy cells per sample (mean +/- standard deviation (SD), n = 3 biological replicates) determined as in A-C. (**D**, **E**) HUVEC (**D**) or U937 (**E**) were exposed to wild-type *L*. *monocytogenes* (wt, JAT115), *L*. *innocua* (Li, JAT638) or *hly L*. *monocytogenes* (*hly*, JAT314). (**D**) Multiplicity of infection (MOI) wt: 5.4, L.i.: 5.4, *hly*: 9.6. (**E**) MOI wt: 6.3, L.i.: 4.5, *hly*: 9.7. (**F, G**) Dose-response of HUVEC (**F**) or U937 (**G**) survival as a function of concentration of purified 6-His-LLO. Insets: Same data, with number of live cells plotted as a function of log([6-His-LLO]). (**H**) HUVEC survival as a function of time. Cells were exposed to wt (JAT115), *hly* (JAT314), or LLO^G486D^ *L*. *monocytogenes* (JAT745) and fixed at successive time-points after infection.
(TIF)

S2 Fig LLO^G486D^ supports vacuolar escape, bacterial replication and cell-to-cell spread. (**A-D**) Time-dependent spread of bacteria in an endothelial monolayer. HUVEC were exposed to JAT983 in a gentamicin protection assay. Samples were fixed 4, 6, 8, 10, or 12 hours after infection and percent of HUVEC infected was quantified by microscopy. (**A, B**) Representative images from (**A**) 4 or (**B**) 12 hours after infection. Blue: HUVEC nuclei. Black: *L*. *monocytogenes*. Scale bars: 100μm. (**C**) Percent of HUVEC infected increased exponentially with time (mean +/- SD, n = 16 biological replicates). (**D**) Growth in focus size as a function of time was more dramatic for the largest decile of foci (mean +/- SD, n = 16 biological replicates). (**E-L**) HUVEC were infected with JAT983 or JAT 985, and analyzed by microscopy 8 hours after infection. (**E, F**) Representative images from HUVEC infected with JAT983(LLO^G486D^) (**E**) or JAT985 (LLO^G486D^ *actA*) (**F**). Blue: HUVEC nuclei. Black: *L*. *monocytogenes*. Scale bars: 100μm. (**G**) Fraction of HUVEC infected with JAT983(LLO^G486D^) versus JAT985 (LLO^G486D^ *actA*) (mean +/- SD, n = 4 biological replicates). (**H**) Number of foci (mean +/- SD, n = 4 biological replicates). (**I**) Mean density of bacteria per infected cell (mean +/- SD, n = 4 biological replicates). (**J**) Distribution of bacterial density per infected cell for JAT983(LLO^G486D^) (n = 593 cells) and JAT985 (LLO^G486D^ *actA*) (n = 295). (**K**) Focus size (mean +/- SD, n = 4 biological replicates). (**L**) Distribution of focus size for JAT983(LLO^G486D^) (n = 122 foci) and JAT985 (LLO^G486D^ *actA*) (n = 110).
(TIF)

S3 Fig Cell density correlates with number of foci but not with other morphological metrics of infection. HUVEC were seeded at 1250, 2500, 5000 or 10000 cells per well, infected with JAT983, and analyzed by microscopy 8 hours after infection. Each point represents an independent sample. (**A**) Frequency of infection is uncorrelated with the number of cells in the sample. (**B**) Number of foci is linearly correlated with number of cells. (**C**) Density of bacteria per infected cell is uncorrelated with number of cells. (**D**) Focus size is uncorrelated with number of cells.
(TIF)

S4 Fig Infected cells can be identified by flow cytometry. Single cells are identified as in S1A–S1C Fig (**A**) The singlet population of an unexposed sample is visualized on a plot of the *L*. *monocytogenes* fluorescence channel versus a non-specific fluorophore that is used as a proxy for cellular autofluorescence. The green gate to define infected cells is drawn to exclude nearly all of the cells in the unexposed sample. (**B**) In the singlet population of a sample exposed to bacteria, many cells fall into the gate that defines infected cells. (**C**) In a histogram of intensity of the bacterial fluorescence channel, the unexposed single cells exhibit a single low-fluorescence peak. An exposed sample reveals two peaks, corresponding to the infected and uninfected cells in the sample. The gate for infected cells produces a population with a single high fluorescence peak. (**D**) Schematic of the drug addition experiments. Top: Gentamicin protection. Middle: If the drug is present prior to gentamicin addition, it will have an effect if the target affects bacterial uptake. Bottom: If the drug is added with gentamicin, it will have an effect if the target affects infection after uptake.
(TIF)

S5 Fig Arp2/3 complex depletion affects cell-to-cell spread but not invasion. (**A**,**B**) HUVEC were treated with synthetic siRNA pools to *ACTR2* (green), or control (blue), infected with JAT983 and analyzed by microscopy 8 hours after infection. (**A**) Frequency of infected HUVEC is comparable for control and siACTR2-treated cells across a range of bacterial doses (mean +/- SD, n = 8 biological replicates). (**B**) Bacterial density per infected cell is higher for siACTR2-treated cells than for controls (mean +/- SD, n = 8 biological replicates). (**C**, **D**) HUVEC in which *ARPC2* (encoding the Arp2/3 complex subunit p34) is depleted exhibit a phenotype consistent with impaired cell-to-cell spread. HUVEC were treated with control siRNAs (**C**) or siRNAs targeting *ARPC2* (**D**), and infected with *L*. *monocytogenes* (JAT983). Samples were fixed and stained with phalloidin 3.5 hours after infection. (i) Intracellular bacteria (expressing RFP) (ii) Polymerized actin (labeled with AF488-phalloidin) (iii) In overlay, actin is associated with bacteria in the control sample (**C**, iii) but not in the ARPC2-depleted sample (**D**, iii). (iv) Phase-contrast image of the same region. Scale bars: 5μm. (**E**) HUVEC were treated with synthetic siRNA pools targeting *ARPC2* or *ACTR2*, control siRNA pools, or not treated with siRNA. Knockdown was performed in triplicate. Samples were lysed, run on an SDS-PAGE gel, and total protein (left) demonstrated comparable loading of samples. Western blot for Arp2 (right) shows complete depletion in the siARPC2- and siACTR2-treated samples but not in the control samples, as expected given previous studies showing that the entire Arp2/3 complex is destabilized by depletion of individual subunits [93,94].
(TIF)

S6 Fig Validation of gene expression changes after siRNA treatment. Relative expression obtained by qPCR, analyzed as described in *Materials and Methods*. Expression of the gene of interest in the siRNA treated sample relative to the control-treated sample (NRQ) is presented as an average ± SD of three biological replicates. (**A**) Key siRNA pools from the Dicer library. (**B**) Synthetic siRNA pools. (**C**) Synthetic siRNA pools targeting FMNL3, INF2, or GRID2IP do not decrease expression of FHOD1. (**D**) Synthetic siRNA pools targeting FHOD1, INF2, or GRID2IP do not decrease expression of FMNL3.
(TIF)

S7 Fig FAK inhibitors promote large focal adhesions in HUVEC. Focal adhesions were visualized with a paxillin antibody (left column, green in overlay). Phalloidin (red in overlay) and DAPI (blue in overlay) were used to visualize cells. (**A**) Cells were treated with vehicle control (DMSO). Large focal adhesions are not visible. (**B**) Cells were treated with 5uM FAK-14. Yellow arrows indicate some large focal adhesions.
(TIF)

S1 Movie *L*. *monocytogenes* LLO^G486D^ has invaded HUVEC, escaped the vacuole, and is moving within the cytoplasm.100X real time. (MOV)

S2 Movie Endothelial cells infected with *L*. *monocytogenes* LLO^G486D^ (JAT983, superimposed in green). Numerous bacteria are moving throughout the cytoplasm and forming protrusions. 100X real time.
(MOV)

S3 Movie HUVEC infected with wild-type (JAT607) *L*. *monocytogenes*. 100X real time.
(MOV)

S4 Movie Successful transfer of *L*. *monocytogenes* LLO^G486D^ (JAT983, superimposed in green) bacterial protrusion from an endothelial cell into an adjacent uninfected cell. 100X real time. Scale bar: 10μM.
(MOV)

S5 Movie Multiple *L*. *monocytogenes* LLO^G486D^ (JAT983, superimposed in green) bacteria can be transferred from an endothelial cell into an adjacent uninfected cell and acquire motility in the newly infected cell. 1800X real time. Scale bar: 50μM.
(MOV)

S1 Table siRNAs in the screening library generated from *in vitro* dicing. (XLS)

S2 Table *actA* screen. (XLSX)

S3 Table Bacterial strains used in this study. (PDF)

S4 Table Synthetic siRNA pools used in this study. (PDF)

S5 Table RTqPCR primers used in this study. (PDF)
