# Supplementary figures and images for: Endothelial Cells Use a Formin-Dependent Phagocytosis-Like Process to Internalize the Bacterium Listeria monocytogenes
Source: PLoS Pathog. 2016 May 6;12(5):e1005603. doi: 10.1371/journal.ppat.1005603 (PMC4859537; doi:10.1371/journal.ppat.1005603)

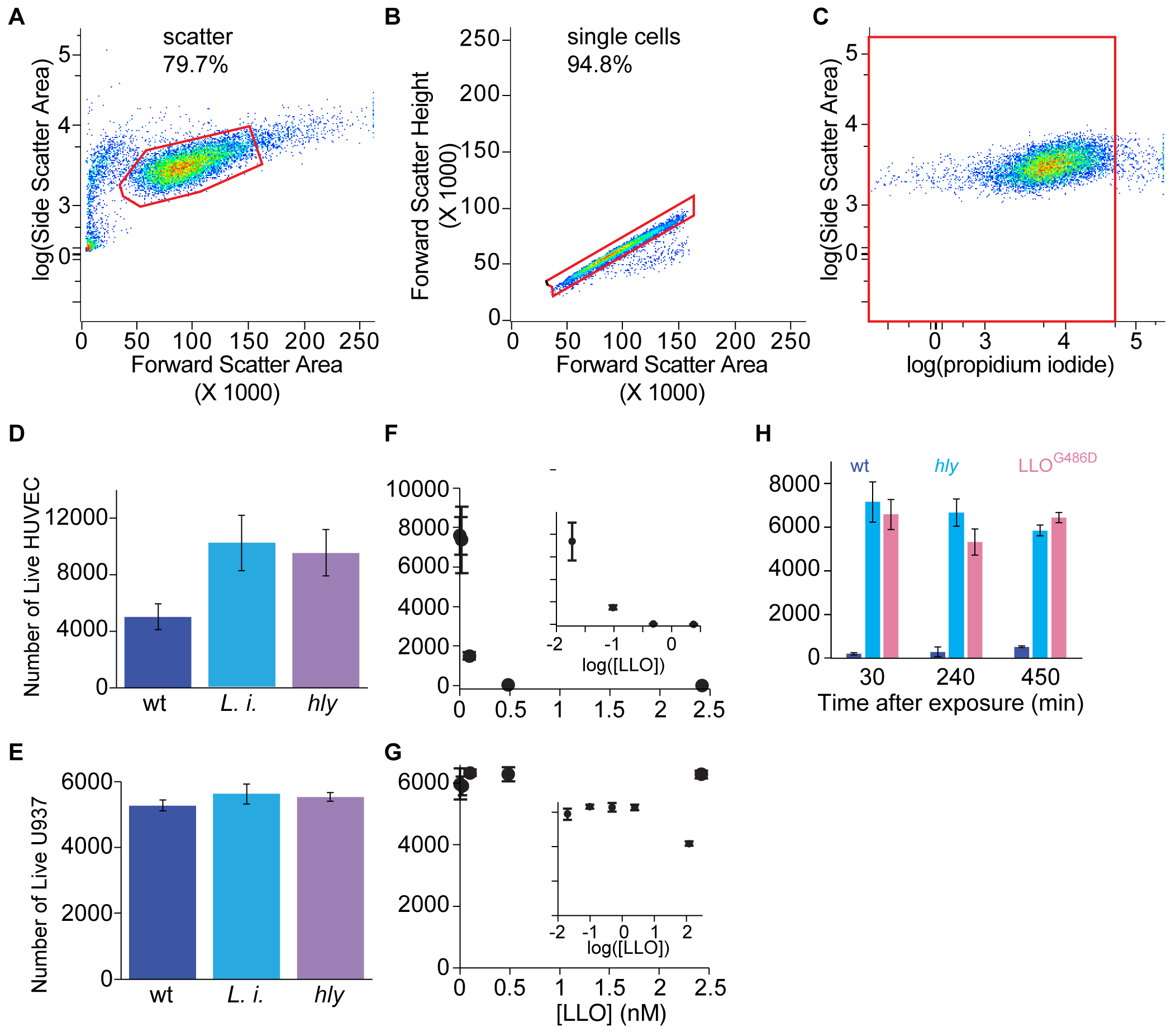

Supplement: S1 Fig — (A-C) Quantification of healthy cells by flow cytometry. (A) Single cells isolated by forward scatter area and side scatter area. The bulk of the distribution in the forward scatter area vs. side scatter area plot (enclosed by the red “scatter” gate) is single cells. (B) Refinement of single cell population using forward scatter height. The contents of the scatter gate (in A) are again gated to collect the bulk of the distribution on the forward scatter area vs. height plot. Outliers are more likely to be doublets or triplets. (C) Isolation of live cells. The contents of the single cell gate (in B) are gated to collect the live cells, which have not taken up propidium iodide. (D-G) Number of healthy cells per sample (mean +/- standard deviation (SD), n = 3 biological replicates) determined as in A-C. (D, E) HUVEC (D) or U937 (E) were exposed to wild-type L. monocytogenes (wt, JAT115), L. innocua (Li, JAT638) or hly L. monocytogenes (hly, JAT314). (D) Multiplicity of infection (MOI) wt: 5.4, L.i.: 5.4, hly: 9.6. (E) MOI wt: 6.3, L.i.: 4.5, hly: 9.7. (F, G) Dose-response of HUVEC (F) or U937 (G) survival as a function of concentration of purified 6-His-LLO. Insets: Same data, with number of live cells plotted as a function of log([6-His-LLO]). (H) HUVEC survival as a function of time. Cells were exposed to wt (JAT115), hly (JAT314), or LLOG486D L. monocytogenes (JAT745) and fixed at successive time-points after infection. (TIF) [file ppat.1005603.s001.tif]

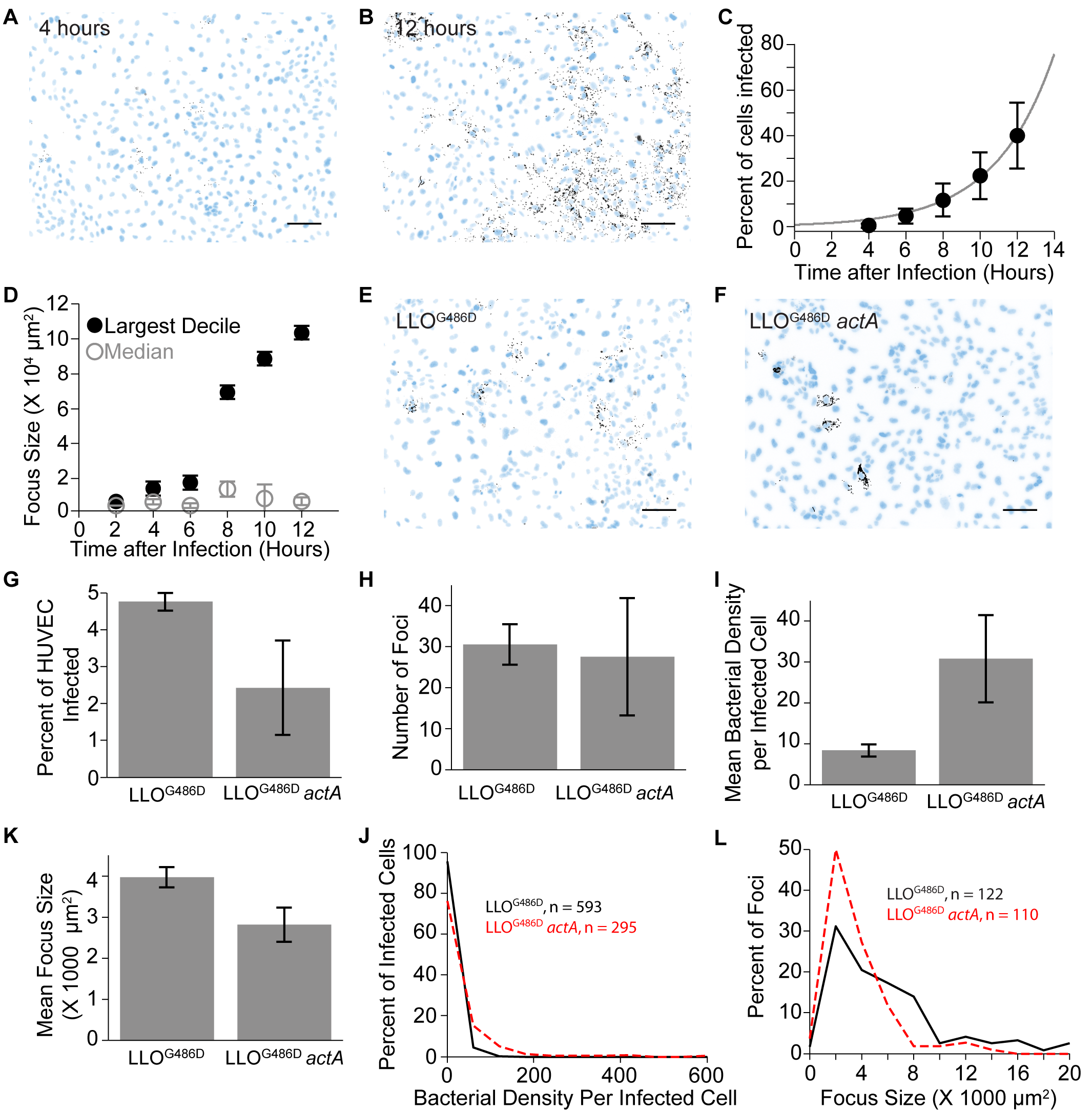

Supplement: S2 Fig — (A-D) Time-dependent spread of bacteria in an endothelial monolayer. HUVEC were exposed to JAT983 in a gentamicin protection assay. Samples were fixed 4, 6, 8, 10, or 12 hours after infection and percent of HUVEC infected was quantified by microscopy. (A, B) Representative images from (A) 4 or (B) 12 hours after infection. Blue: HUVEC nuclei. Black: L. monocytogenes. Scale bars: 100μm. (C) Percent of HUVEC infected increased exponentially with time (mean +/- SD, n = 16 biological replicates). (D) Growth in focus size as a function of time was more dramatic for the largest decile of foci (mean +/- SD, n = 16 biological replicates). (E-L) HUVEC were infected with JAT983 or JAT 985, and analyzed by microscopy 8 hours after infection. (E, F) Representative images from HUVEC infected with JAT983(LLOG486D) (E) or JAT985 (LLOG486D actA) (F). Blue: HUVEC nuclei. Black: L. monocytogenes. Scale bars: 100μm. (G) Fraction of HUVEC infected with JAT983(LLOG486D) versus JAT985 (LLOG486D actA) (mean +/- SD, n = 4 biological replicates). (H) Number of foci (mean +/- SD, n = 4 biological replicates). (I) Mean density of bacteria per infected cell (mean +/- SD, n = 4 biological replicates). (J) Distribution of bacterial density per infected cell for JAT983(LLOG486D) (n = 593 cells) and JAT985 (LLOG486D actA) (n = 295). (K) Focus size (mean +/- SD, n = 4 biological replicates). (L) Distribution of focus size for JAT983(LLOG486D) (n = 122 foci) and JAT985 (LLOG486D actA) (n = 110). (TIF) [file ppat.1005603.s002.tif]

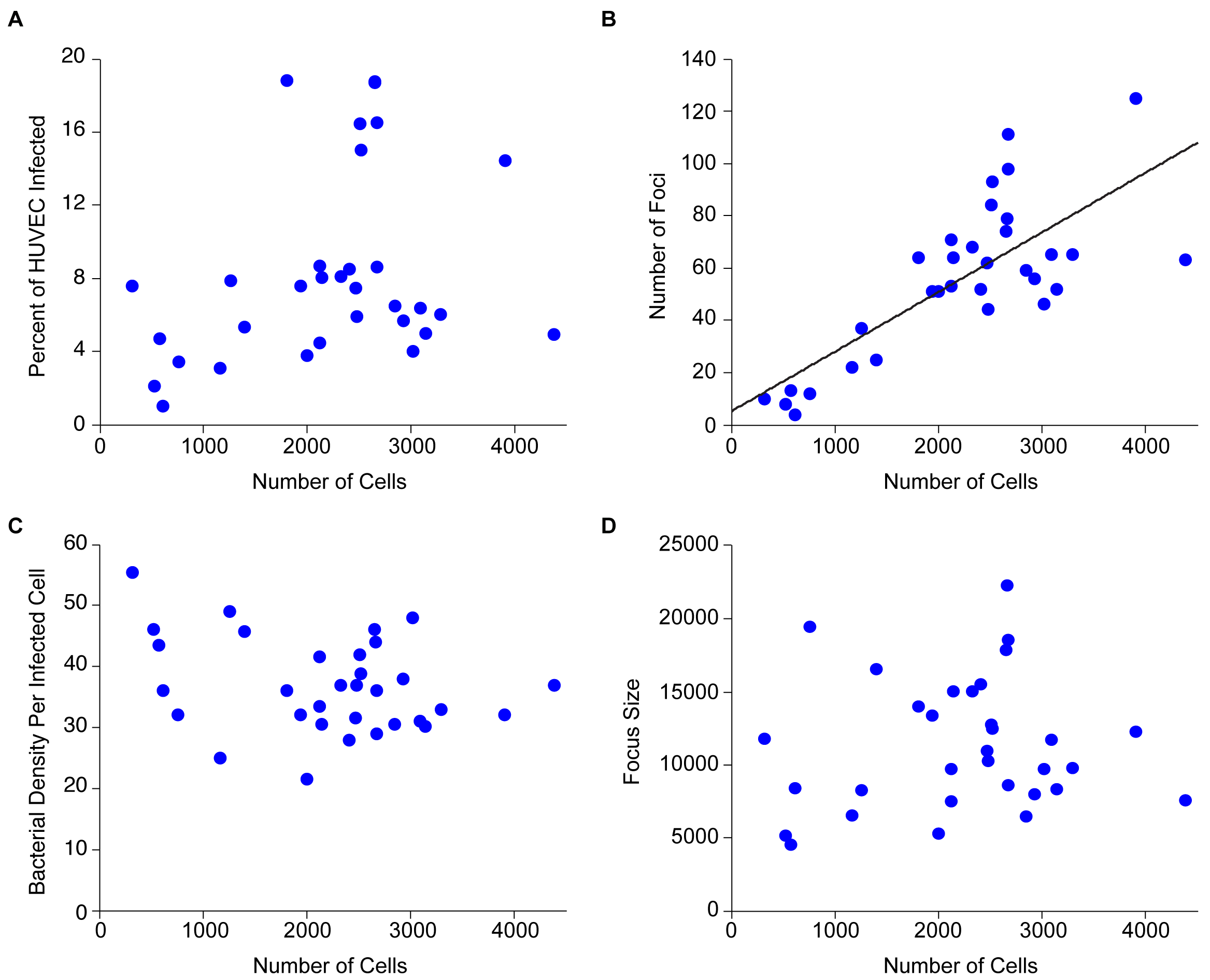

Supplement: S3 Fig — HUVEC were seeded at 1250, 2500, 5000 or 10000 cells per well, infected with JAT983, and analyzed by microscopy 8 hours after infection. Each point represents an independent sample. (A) Frequency of infection is uncorrelated with the number of cells in the sample. (B) Number of foci is linearly correlated with number of cells. (C) Density of bacteria per infected cell is uncorrelated with number of cells. (D) Focus size is uncorrelated with number of cells. (TIF) [file ppat.1005603.s003.tif]

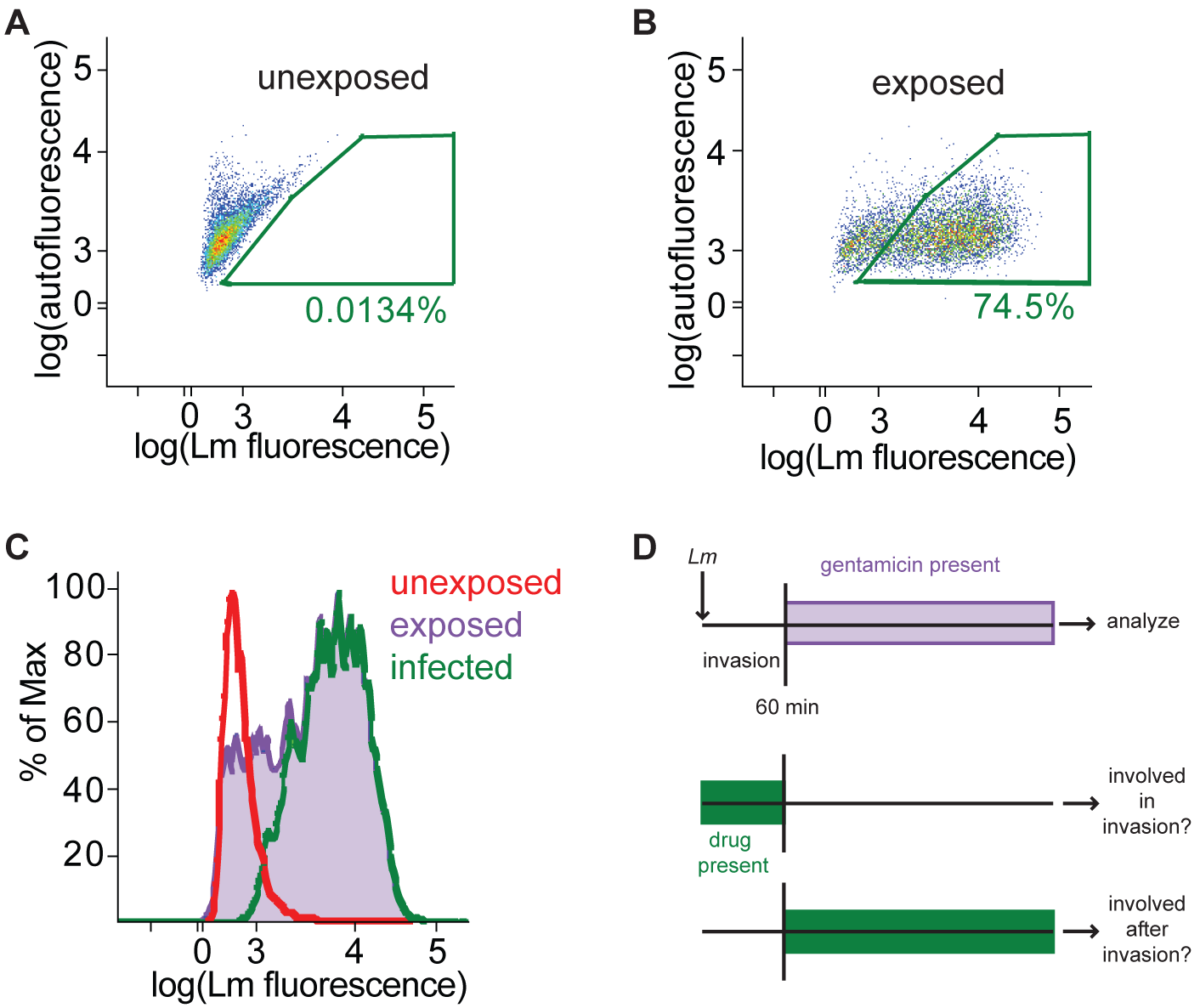

Supplement: S4 Fig — Single cells are identified as in S1A–S1C Fig (A) The singlet population of an unexposed sample is visualized on a plot of the L. monocytogenes fluorescence channel versus a non-specific fluorophore that is used as a proxy for cellular autofluorescence. The green gate to define infected cells is drawn to exclude nearly all of the cells in the unexposed sample. (B) In the singlet population of a sample exposed to bacteria, many cells fall into the gate that defines infected cells. (C) In a histogram of intensity of the bacterial fluorescence channel, the unexposed single cells exhibit a single low-fluorescence peak. An exposed sample reveals two peaks, corresponding to the infected and uninfected cells in the sample. The gate for infected cells produces a population with a single high fluorescence peak. (D) Schematic of the drug addition experiments. Top: Gentamicin protection. Middle: If the drug is present prior to gentamicin addition, it will have an effect if the target affects bacterial uptake. Bottom: If the drug is added with gentamicin, it will have an effect if the target affects infection after uptake. (TIF) [file ppat.1005603.s004.tif]

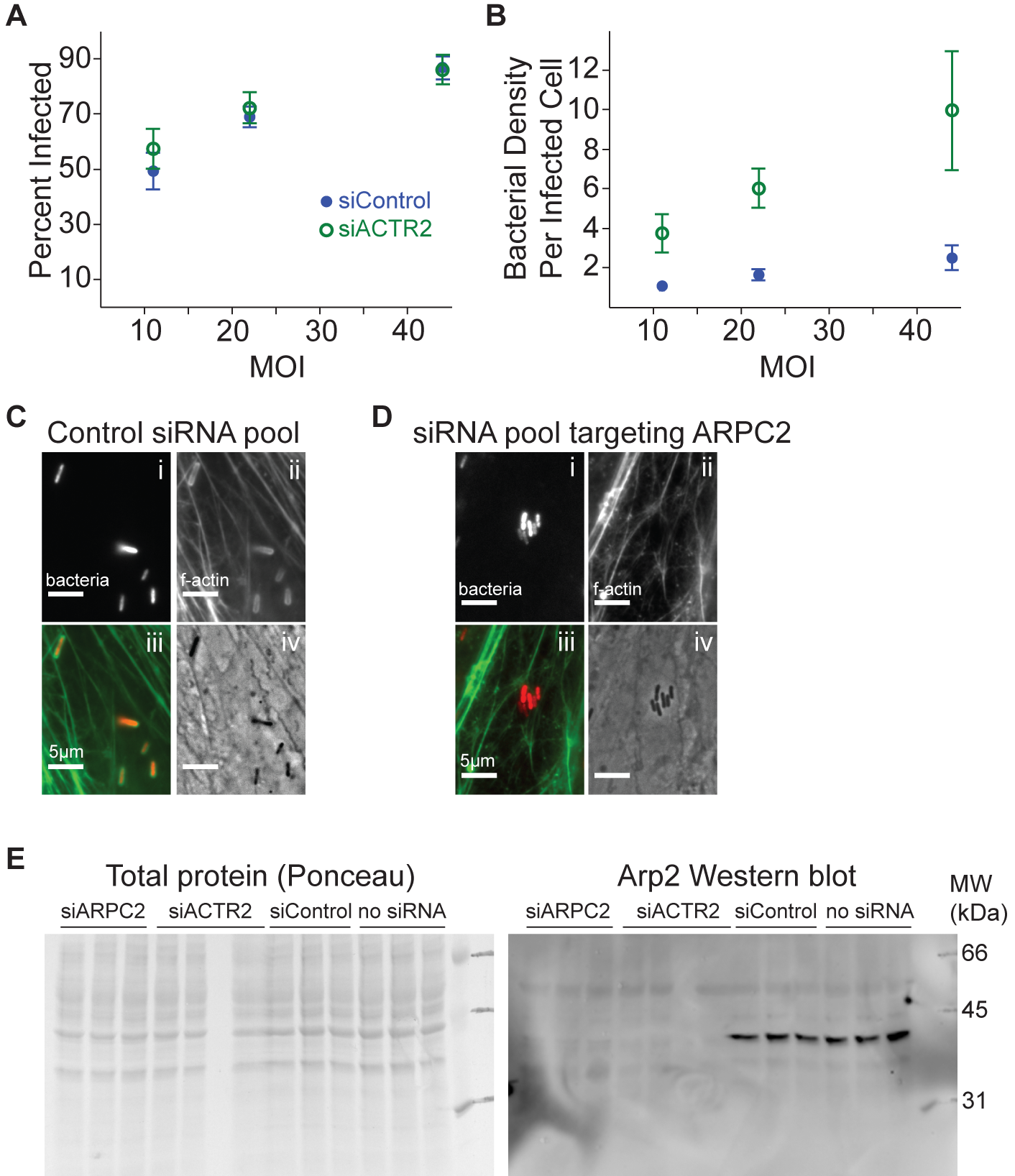

Supplement: S5 Fig — (A,B) HUVEC were treated with synthetic siRNA pools to ACTR2 (green), or control (blue), infected with JAT983 and analyzed by microscopy 8 hours after infection. (A) Frequency of infected HUVEC is comparable for control and siACTR2-treated cells across a range of bacterial doses (mean +/- SD, n = 8 biological replicates). (B) Bacterial density per infected cell is higher for siACTR2-treated cells than for controls (mean +/- SD, n = 8 biological replicates). (C, D) HUVEC in which ARPC2 (encoding the Arp2/3 complex subunit p34) is depleted exhibit a phenotype consistent with impaired cell-to-cell spread. HUVEC were treated with control siRNAs (C) or siRNAs targeting ARPC2 (D), and infected with L. monocytogenes (JAT983). Samples were fixed and stained with phalloidin 3.5 hours after infection. (i) Intracellular bacteria (expressing RFP) (ii) Polymerized actin (labeled with AF488-phalloidin) (iii) In overlay, actin is associated with bacteria in the control sample (C, iii) but not in the ARPC2-depleted sample (D, iii). (iv) Phase-contrast image of the same region. Scale bars: 5μm. (E) HUVEC were treated with synthetic siRNA pools targeting ARPC2 or ACTR2, control siRNA pools, or not treated with siRNA. Knockdown was performed in triplicate. Samples were lysed, run on an SDS-PAGE gel, and total protein (left) demonstrated comparable loading of samples. Western blot for Arp2 (right) shows complete depletion in the siARPC2- and siACTR2-treated samples but not in the control samples, as expected given previous studies showing that the entire Arp2/3 complex is destabilized by depletion of individual subunits [93,94]. (TIF) [file ppat.1005603.s005.tif]

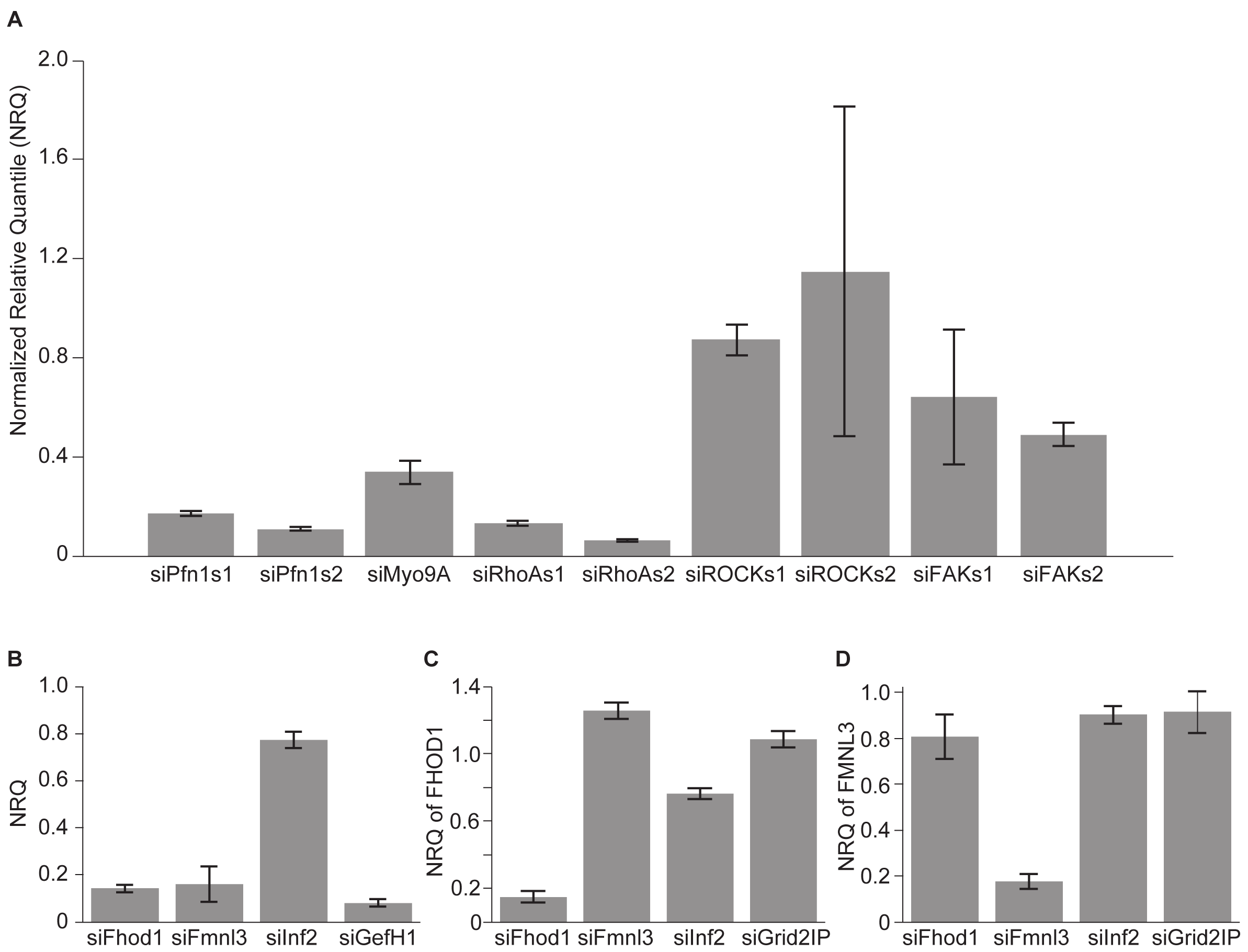

Supplement: S6 Fig — Relative expression obtained by qPCR, analyzed as described in Materials and Methods. Expression of the gene of interest in the siRNA treated sample relative to the control-treated sample (NRQ) is presented as an average ± SD of three biological replicates. (A) Key siRNA pools from the Dicer library. (B) Synthetic siRNA pools. (C) Synthetic siRNA pools targeting FMNL3, INF2, or GRID2IP do not decrease expression of FHOD1. (D) Synthetic siRNA pools targeting FHOD1, INF2, or GRID2IP do not decrease expression of FMNL3. (TIF) [file ppat.1005603.s006.tif]

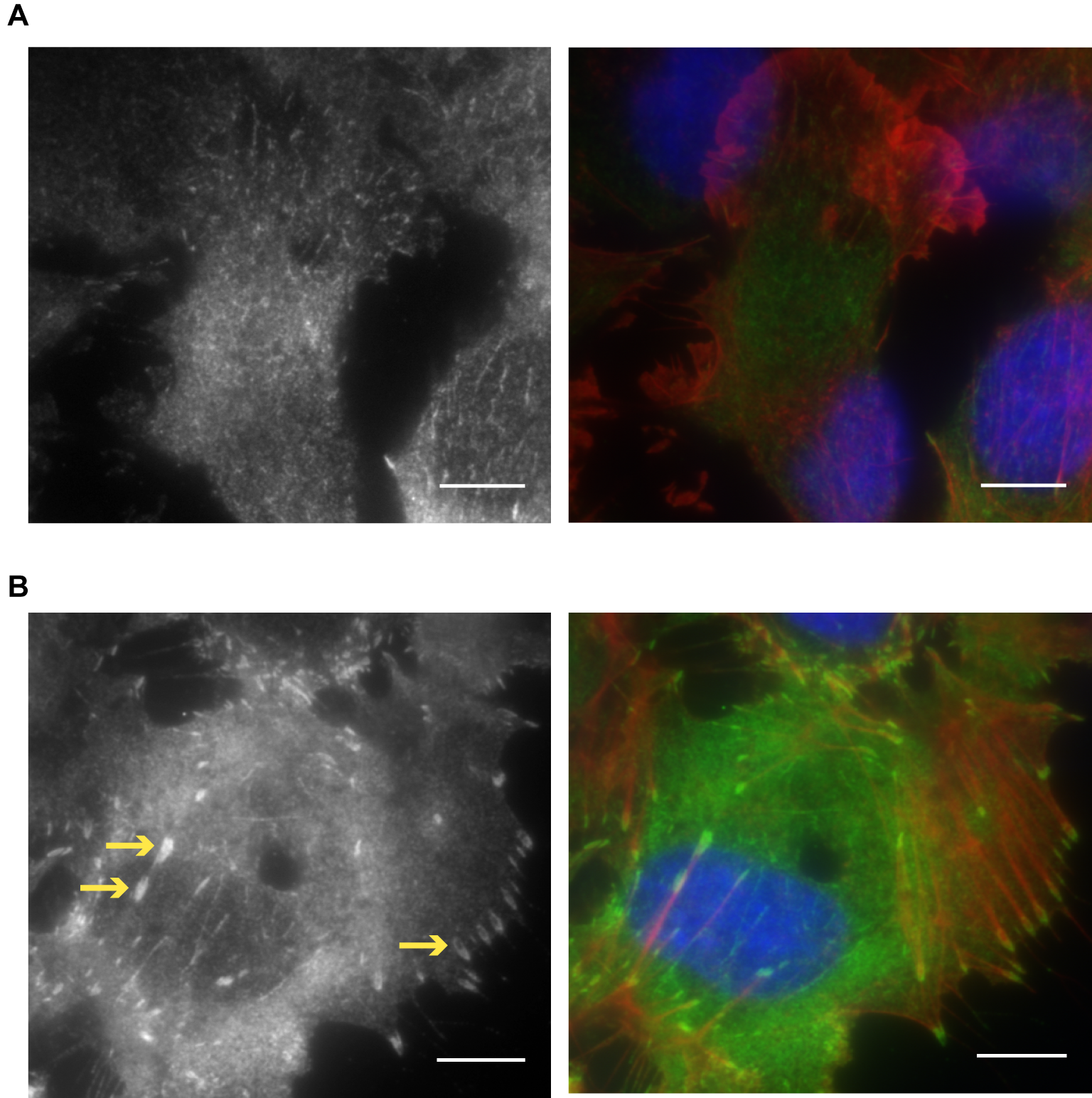

Supplement: S7 Fig — Focal adhesions were visualized with a paxillin antibody (left column, green in overlay). Phalloidin (red in overlay) and DAPI (blue in overlay) were used to visualize cells. (A) Cells were treated with vehicle control (DMSO). Large focal adhesions are not visible. (B) Cells were treated with 5uM FAK-14. Yellow arrows indicate some large focal adhesions. (TIF) [file ppat.1005603.s007.tif]
