# Supplementary material for: Endothelial Cells Use a Formin-Dependent Phagocytosis-Like Process to Internalize the Bacterium Listeria monocytogenes
Source: PLoS Pathog. 2016 May 6;12(5):e1005603. doi: 10.1371/journal.ppat.1005603 (PMC4859537; doi:10.1371/journal.ppat.1005603)
Supplement: S3 Table — (PDF) [file ppat.1005603.s015.pdf]

**Supplementary Table 3: Bacterial strains used in this study**

| Name and source | Species | Genotype/Description |
|---|---|---|
| JAT115 (from Portnoy Lab) | *L. monocytogenes* 10403S | Wild-type |
| JAT314 (DP-L2161)[95] | *L. monocytogenes* 10403S | *hly* |
| JAT638 (DP-L392)[83] | *L. innocua* | |
| JAT745 (DP-L4044)[26] | *L. monocytogenes* 10403S | $LLO^{G486D}$ |
| JAT983 (This study) | *L. monocytogenes* 10403S | $LLO^{G486D}$ actAp::mTagRFP |
| JAT984 (DP-L4560)[27] | *L. monocytogenes* 10403S | $LLO^{G486D}$ *ΔactA* |
| JAT985 (This study) | *L. monocytogenes* 10403S | $LLO^{G486D}$ *ΔactA* actAp::mTagRFP |
| JAT1084 (DP-L4404)[96] | *L. monocytogenes* 10403S | *ΔinlAB* |
| JAT1116 (This study) | *L. monocytogenes* 10403S | $LLO^{G486D}$ *ΔinlAB* |
| JAT1119 (This study) | *L. monocytogenes* 10403S | $LLO^{G486D}$ *ΔinlAB* actAp::mTagRFP |
| JAT1045 (This study) | *L. monocytogenes* 10403S | $LLO^{G486D}$ Constitutive sGFP |
| JAT1046 (This study) | *L. monocytogenes* 10403S | $LLO^{G486D}$ *ΔinlAB* Constitutive sGFP |
| JAT607 (This study, also see others[28]) | *L. monocytogenes* 10403S | 10403S actAp::mTagRFP |
| JAT610 (This study) | *L. monocytogenes* 10403S | 10403S *ΔactA* actAp::mTagRFP |
| JAT602 (This study, also see others[85]) | *E. coli* SM10 | Carries plasmid pPL499 |
| JAT609 (This study, also see others[85]) | *E.coli* SM10 | Carries plasmid pMP74 |
| JAT1223 (This study, DP-E4043 (Portnoy Lab), carries the plasmid in DH5a) | *E. coli* SM10 | Carries plasmid pCON1 with the $LLO^{G486D}$ point mutation |

95. Jones S, Portnoy DA (1994) Characterization of *Listeria monocytogenes* pathogenesis in a strain expressing perfringolysin O in place of listeriolysin O. Infect Immun 62: 5608-5613.

96. Bakardjiev AI, Theriot JA, Portnoy DA (2006) *Listeria monocytogenes* traffics from maternal organs to the placenta and back. PLoS Pathog 2: e66.
