# Supplementary material for: Endothelial Cells Use a Formin-Dependent Phagocytosis-Like Process to Internalize the Bacterium Listeria monocytogenes
Source: PLoS Pathog. 2016 May 6;12(5):e1005603. doi: 10.1371/journal.ppat.1005603 (PMC4859537; doi:10.1371/journal.ppat.1005603)
Supplement: S4 Table — (PDF) [file ppat.1005603.s016.pdf]

Supplementary Table 4: Synthetic siRNA pools used in this study.

Accession Number	Gene (Protein)	Product Name	Catalog Number
	Negative Control	ON-TARGETplus Non-Targeting Pool	D-001810-10
NM_001005386	*ACTR2* (Arp2)	SMARTpool: siGENOME ACTR2 siRNA	M-012076-01
NM_001173	*ARHGAP5* (p190RhoGAP)	SMARTpool: ON-TARGETplus Human ARHGAP5 siRNA	L-009580-00
NM_001162383	*ARHGEF2* (GEFH1)	SMARTpool: ON-TARGETplus Human ARHGEF2 siRNA	L-009883-00
NM_152862, NM_005731	*ARPC2* (p34-Arc)	SMARTpool: siGENOME ARPC2 siRNA	M-012081-00
NM_005219	*DIAPH1*	SMARTpool: ON-TARGETplus Human DIAPH1 siRNA	L-010347-00
NM_007309	*DIAPH2*	SMARTpool: ON-TARGETplus Human DIAPH2 siRNA	L-012029-00
NM_030932	*DIAPH3*	SMARTpool: ON-TARGETplus Human DIAPH3 siRNA	L-018997-00
NM_005892	*FMNL1*	SMARTpool: ON-TARGETplus Human FMNL1 siRNA	L-019176-00
NM_052905	*FMNL2*	SMARTpool: ON-TARGETplus Human FMNL2 siRNA	L-031993-01
NM_198900	*FMNL3*	SMARTpool: ON-TARGETplus Human FMNL3 siRNA	L-019007-02
NM_014992	*DAAM1*	SMARTpool: ON-TARGETplus Human DAAM1 siRNA	L-012925-00
NM_015345	*DAAM2*	SMARTpool: ON-TARGETplus Human DAAM2 siRNA	L-014010-00
NM_013241	*FHOD1*	SMARTpool: ON-TARGETplus Human FHOD1 siRNA	L-013709-01
NM_025135	*FHOD3*	SMARTpool: ON-TARGETplus Human FHOD3	L-023411-01

		siRNA	
XM_375185	*FMN1*	SMARTpool: ON-TARGETplus Human FMN1 siRNA	L-030385-00
NM_020066	*FMN2*	SMARTpool: ON-TARGETplus Human FMN2 siRNA	L-022134-02
NM_001145118	*GRID2IP*	SMARTpool: ON-TARGETplus Human GRID2IP siRNA	L-029054-01
NM_033393	*FHDC1*	SMARTpool: ON-TARGETplus Human FHDC1 siRNA	L-024060-02
NM_001031714	*INF2*	SMARTpool: ON-TARGETplus Human INF2 siRNA	L-014097-02
