# Supplementary material for: Endothelial Cells Use a Formin-Dependent Phagocytosis-Like Process to Internalize the Bacterium Listeria monocytogenes
Source: PLoS Pathog. 2016 May 6;12(5):e1005603. doi: 10.1371/journal.ppat.1005603 (PMC4859537; doi:10.1371/journal.ppat.1005603)
Supplement: S5 Table — (PDF) [file ppat.1005603.s017.pdf]

Supplementary Table 5: RTqPCR primers used in this study.

Gene of Interest	Forward Primer	Reverse Primer	Primer Bank ID
ACTR2	AACCTCGGCAGTATTAGAAAGGA	CCTCTCCGATGGGTATCATGTG	154354973c1
CDH5	AAGCGTGAGTCGCAAGAATG	TCTCCAGGTTTTCGCCAGTG	166362712c2
FHOD1	GGGTCAACGCTATCTTGGAAA	CAGCCCCTCTGAATGCACAA	118572598c2
FMNL3	AGTACGGATTCAACCTGGTCA	GCAGCTCCTTGCATACCTCTTT	119120873c2
GAPDH-1	GGAGCGAGATCCCTCCAAAAT	GGCTGTTGTCATACTTCTCATGG	378404907c1
ARHGEF2 (GEFH1)	CAGGCATGACCATGTGCTATG	TTTACAGCGGTTGTGGATAGTC	253735771c1
GRID2IP	AGCTTCGGCTTCACACTTCG	TCCAGAGAATCGGAGTCACTG	223278402c1
GRID2IP	GAAGCCGCGATACTGACCG	CCGCTCATTGGTTACGAAGC	223278402c3
INF2	GTGGAACATTTTCCGCGAGTA	GCCCATACAAAGGCGTTCG	151101234c1
MYH9	CAGCAAGCTGCCGATAAGTAT	CTTGTCGGAAGGCACCCAT	
MYO9A	AAGTGGAGAGGACTACCGCTT	TGTTACCCGTAGCCATGACTG	365192551c1
PFN1	TCAAGTTTTTACGTGAATGGGCT	CGAAGATCCATGCTAAATTCCCC	16753213c2
PTK2 (FAK)	TGGTGCAATGGAGCGAGTATT	CAGTGAACCTCCTCTGACCG	313851041c2
RHOA	GGAAAGCAGGTAGAGTTGGCT	GGCTGTCGATGGAAAAACACAT	50593005c2
ROCK1	AAGTGAGGTTAGGGCGAAATG	AAGGTAGTTGATTGCCAACGAA	112382209c3
